# Supplementary material for: Phytochrome B stabilizes the KNOX transcription factor BP/KNAT1 to promote light-initiated seed germination in Arabidopsis thaliana
Source: Plant Commun. 2025 Sep 4;6(11):101517. doi: 10.1016/j.xplc.2025.101517 (PMC12785155; doi:10.1016/j.xplc.2025.101517)
Supplement: Document S1. Supplemental Figures 1–18 and Supplemental Tables 1 and 2 [file mmc1.pdf]

Plant Communications, Volume *6*

Supplemental information

Phytochrome B stabilizes the KNOX transcription factor BP/KNAT1 to promote light-initiated seed germination in *Arabidopsis thaliana*

Dachuan Gu, Yahan Wang, Minglei Zhao, Hangcong Chen, Shuhua Wu, Xia Jin, Ling Deng, Rujun Ji, Jingyan Xu, Feng Zheng, and Xuncheng Liu

Supplemental Information

Phytochrome B stabilizes the KNOX transcription factor BP/KNAT1 to promote light-initiated seed germination in *Arabidopsis thaliana*

Dachuan Gu[1,2,5], Yahan Wang[1,2,5], Minglei Zhao[3,5], Hangcong Chen[1,2], Suhua Wu[4], Xia Jin[1,2], Ling Deng[1,2], Rujun Ji[1,2], Jinyan Xu[1,2], Feng Zheng[1], Xuncheng Liu[1,2,*]

[1]Guangdong Provincial Key Laboratory of Applied Botany, Key Laboratory of South China Agricultural Plant Molecular Analysis and Genetic Improvement, South China Botanical Garden, Chinese Academy of Sciences, Guangzhou, 510650, China
[2]College of Life Sciences, University of Chinese Academy of Sciences, Beijing, 100049, China
[3]College of Horticulture, South China Agricultural University, Guangzhou, China
[4]Guangdong Provincial Key Laboratory of Tea Plant Resources Innovation and Utilization, Tea Research Institute, Guangdong Academy of Agricultural Sciences, Guangzhou 510640, China
[5]These authors contributed equally to this work

[*]Corresponding author: Xuncheng Liu (xunchengliu@scbg.ac.cn)

Running title: phyB stabilizes BP/KNAT1 to promote seed germination

Short summary: KNOX-type transcription factor BP/ KNAT1 plays a positive role in light-dependent seed germination. BP directly represses the expression of ABA biosynthetic genes *NCED6/9*. The red/far-red light photoreceptor phyB interacts with and stabilizes BP in imbibed seeds. This work reveals that phyB-BP-NCED6/9 cascade promotes light-initiated seed germination by repressing ABA biosynthesis.

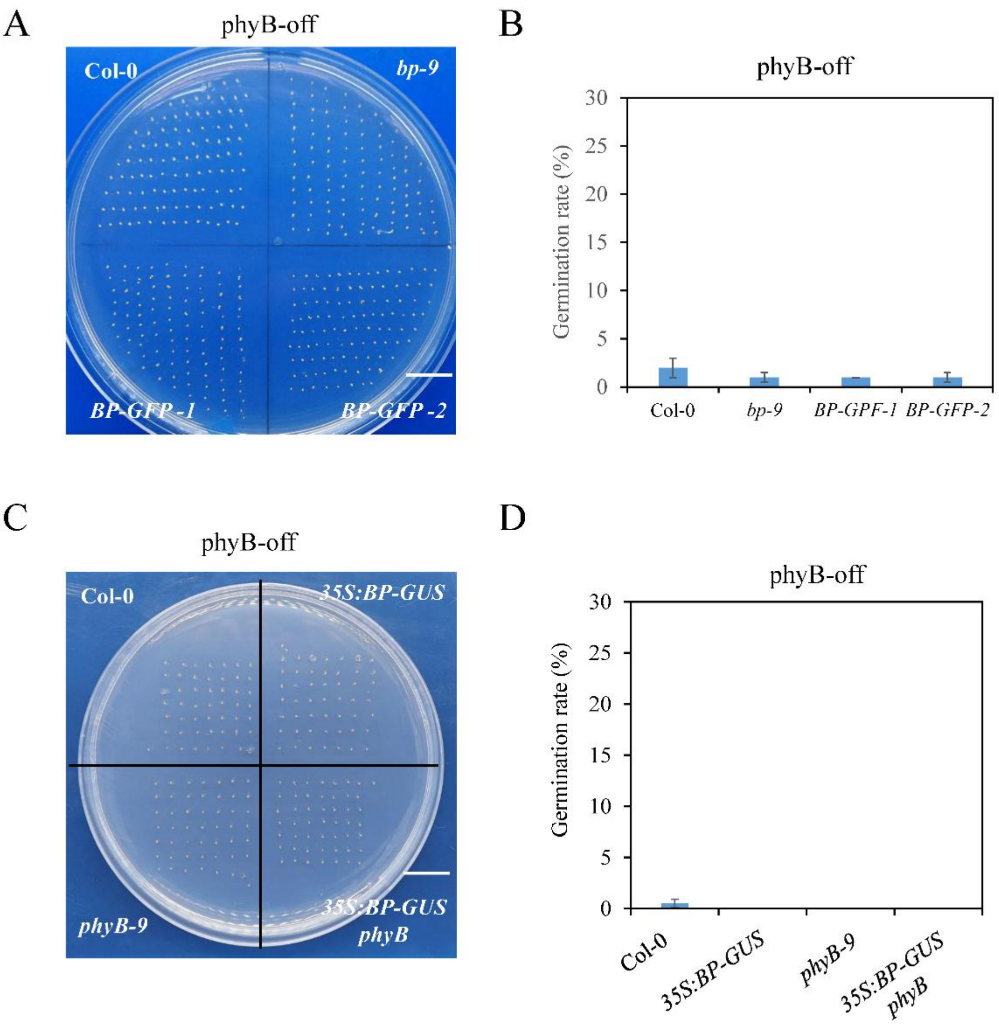

Supplemental Figure 1. Germination phenotype of the *bp-9* mutant, *BP-GFP* complementary lines, *35S:BP-GUS*, and *35S:BP-GUS phyB-9* seeds in the phyB-off condition. (A, B) Germination patterns of Col-0, the *bp-9* mutant, and *BP* transgenic complement lines (*BP-GFP-1* and *-2*) in the phyB-off condition. (C, D) Germination patterns and rates of Col-0, *35S:BP*, *phyB-9*, and *35S:BP phyB* seeds in the phyB-off condition. About 50 seeds of each sample were used for calculations. The experiment was performed in triplicate. Scale bar: 1 cm.

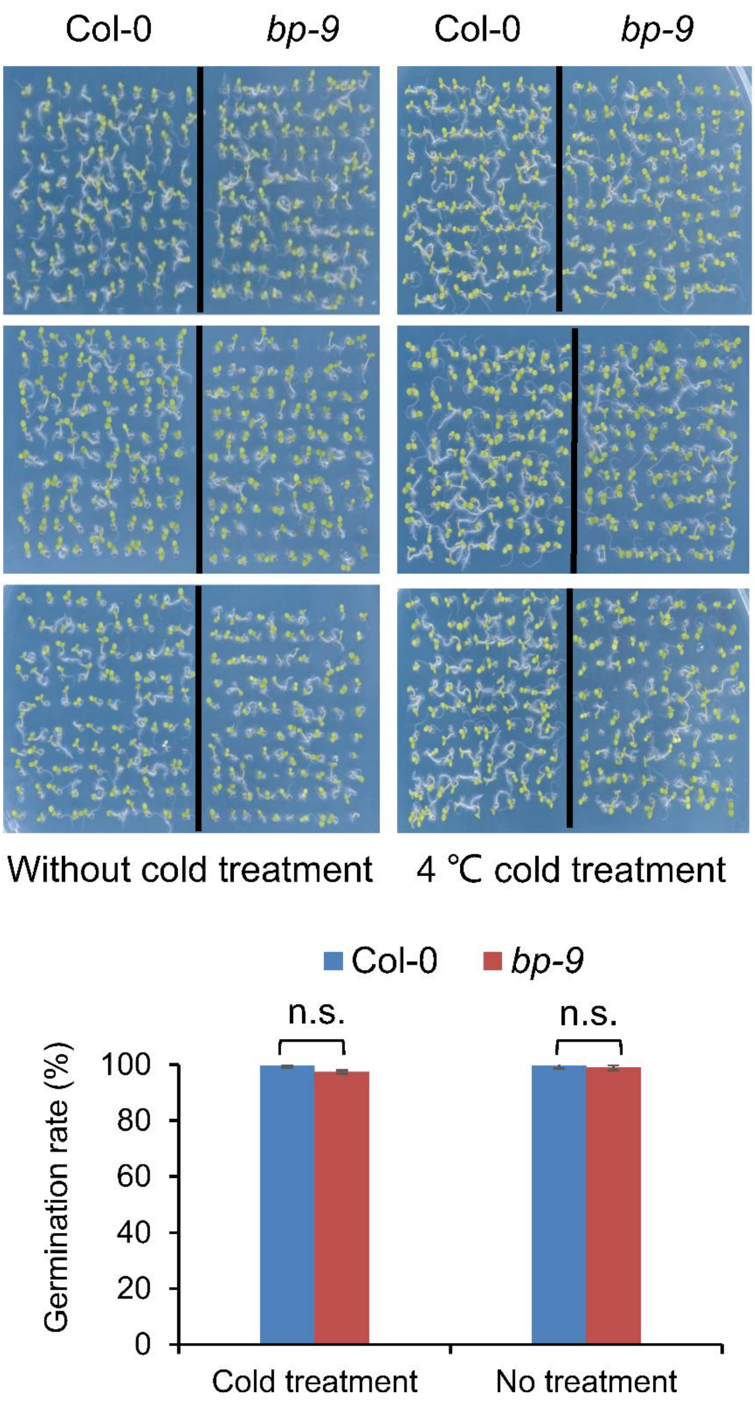

Supplemental Figure 2. Analysis of the effect of stratification treatment (or not) on the germination of *bp-9* mutant. The freshly harvested seeds of Col-0 and *bp-9* were surface sterilized and plated on 0.6% (w/v) agar (pH 5.7) within 1 h. Seeds were either stratified or not at 4 °C for 4 d, followed by incubation in white light (80 μmol $m^{-2}s^{-1}$) for 4 d and scoring of the germination rates. Values are shown as means ± SD (Student's *t*-test, * $p < 0.05$, n=3), n.s. indicate no significant difference.

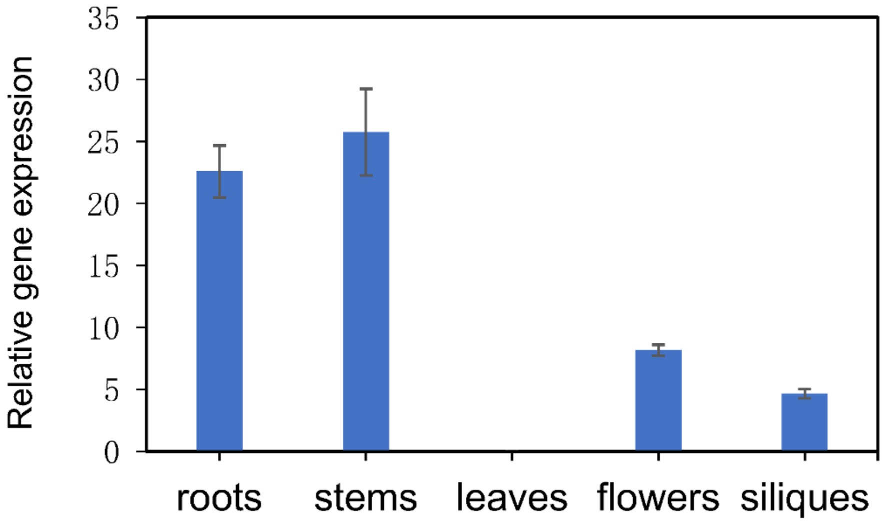

Supplemental Figure 3. RT-qPCR analysis of the transcription levels of *BP* in multiple tissues including roots, stems, leaves, flowers, and siliques of 35-day-old plants. *PP2A* was used as internal control. Values are shown as means ± SD (n=3).

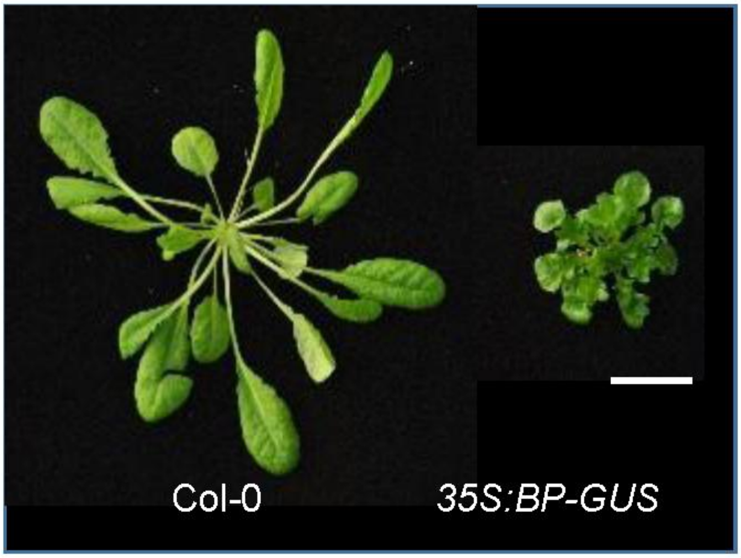

Supplemental Figure 4. Phenotypes of 20-day-od *35S:BP-GUS* transgenic line. Scale bar, 2cm.

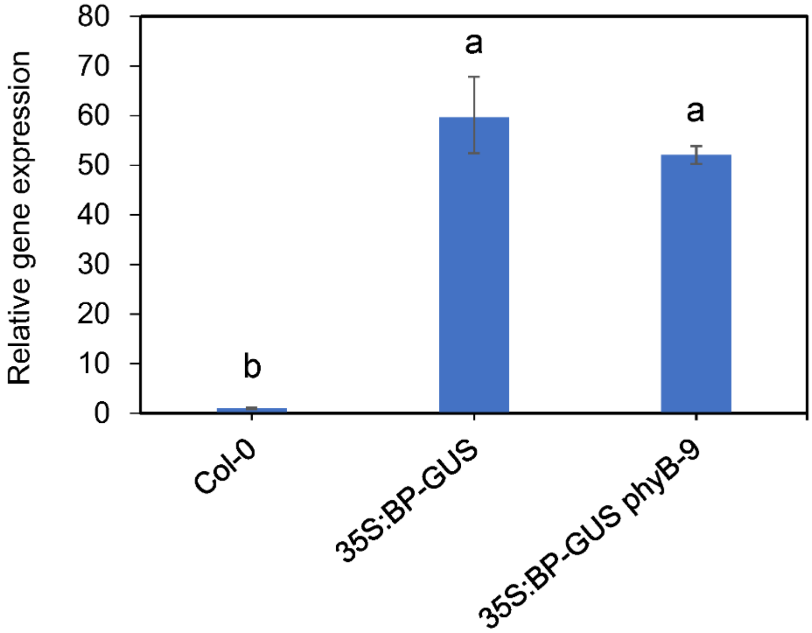

Supplemental Figure 5. RT-qPCR analysis of the expression levels of *BP* in imbibed Col-0, *35S:BP-GUS* and *35S:BP-GUS phyB-9* seeds under phyB-on conditions. *PP2A* was used as internal control. Values are shown as means ± SD. The data were analyzed by one-way ANOVA followed by Tukey's HSD test. Different letters above bars indicate significant differences ($p < 0.05$).

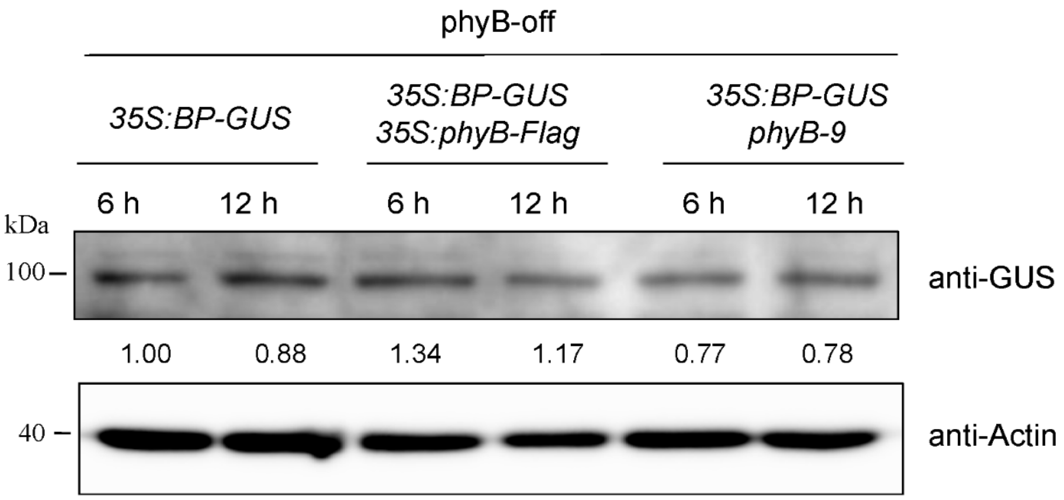

Supplemental Figure 6. Immunoblot analysis to assess the levels of BP protein in *35:BP-GUS*, *35S:BP-GUS 35S:phyB-Flag*, and *35S:BP-GUS phyB-9* seeds in the phyB-off condition. *35S:BP-GUS* and *35S:BP-GUS phyB-9* seeds were treated with FR, then incubated in the dark for 6 or 12 h, respectively. Total proteins were blotted with anti-GUS antibody. Values, which represent relative signal intensity, were normalized to the Actin control.

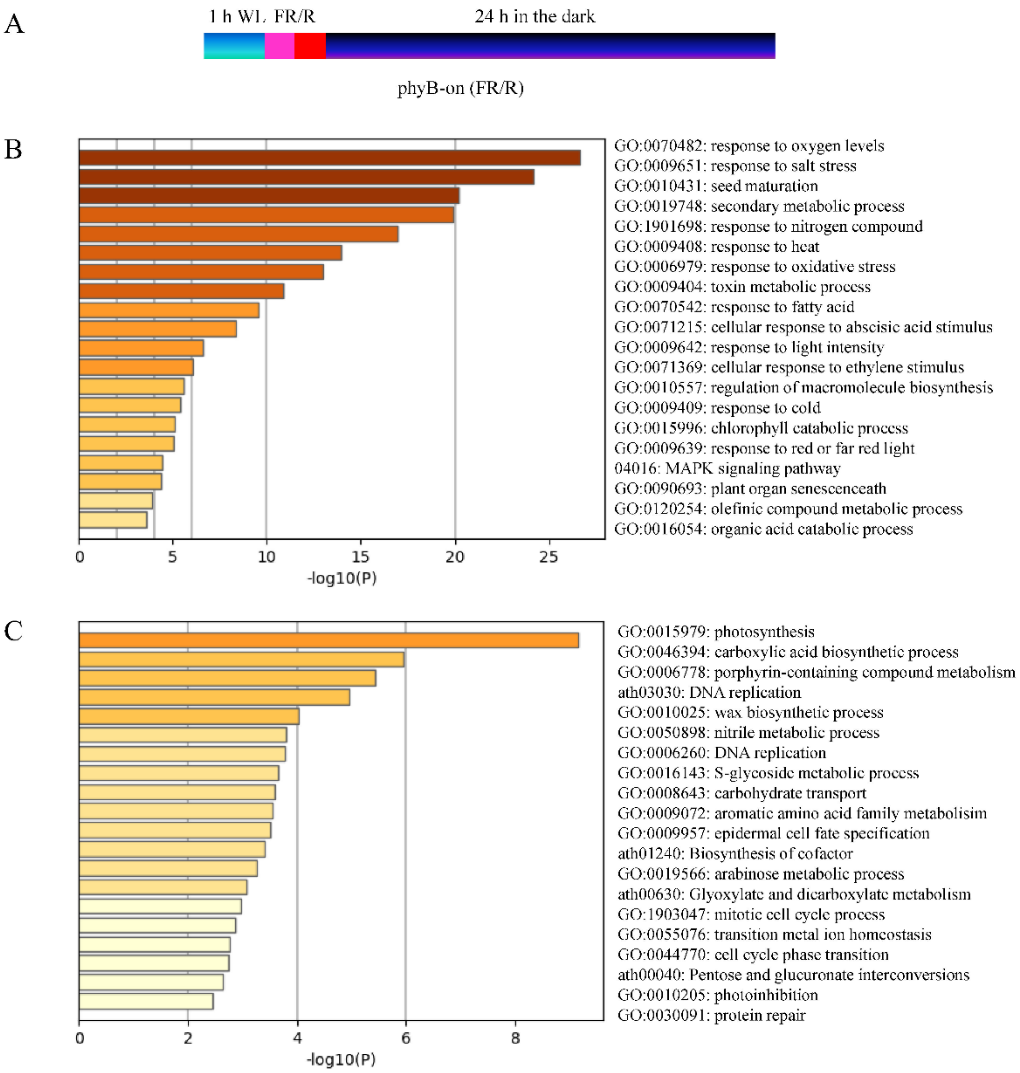

Supplemental Figure 7. Genome-wide analysis of the BP-regulated transcriptome. (A) Diagram of the phyB-on condition. FR/R, 5 min of far-red light (3.8 μmol m^{-2} s^{-1}) and then 5 min of red light (13.1 μmol m^{-2} s^{-1}). After FR/R treatment, the seeds were kept in the dark for 24 h. (B) GO biological process of the up-regulated genes in the *bp-9* mutant, as analyzed by Metascape ($P < 0.01$). (C) GO biological process of the down-regulated genes in the *bp-9* mutant, as analyzed by Metascape ($p < 0.01$).

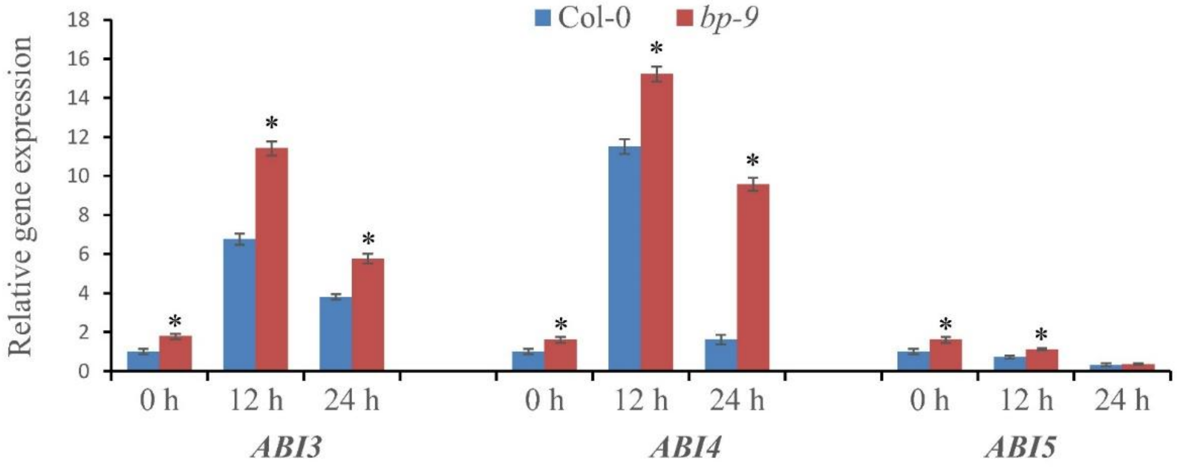

Supplemental Figure 8. RT-qPCR analysis of the expression levels of ABA signal transduction-related genes *ABI3*, *ABI4*, and *ABI5* in the *bp-9* mutant in the phyB-on condition. *PP2A* was used as the internal control. Values are shown as means ± SD (Student's *t*-test, * $p < 0.05$, n = 3).

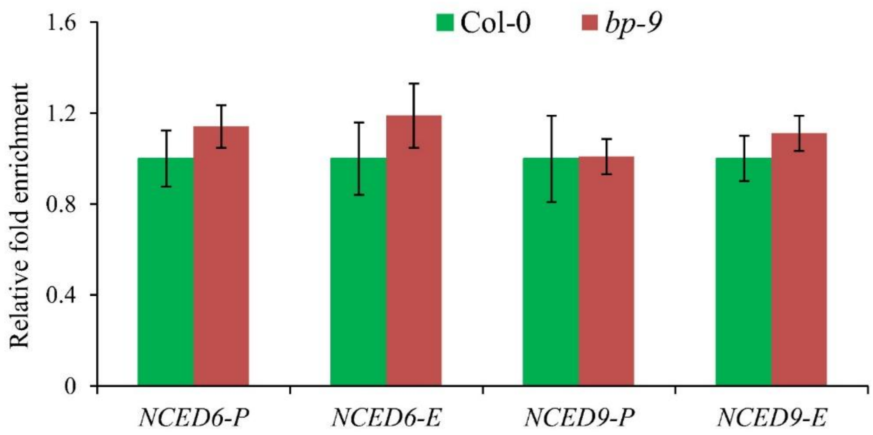

Supplemental Figure 9. ChIP-qPCR analysis of the H3K4me3 levels of *NCED* genes in imbibed Col-0 and *bp-9* mutant seeds. *ACTIN2* was used as the internal control. Values are shown as means ± SD (Student's *t*-test, * $p < 0.05$, n = 3).

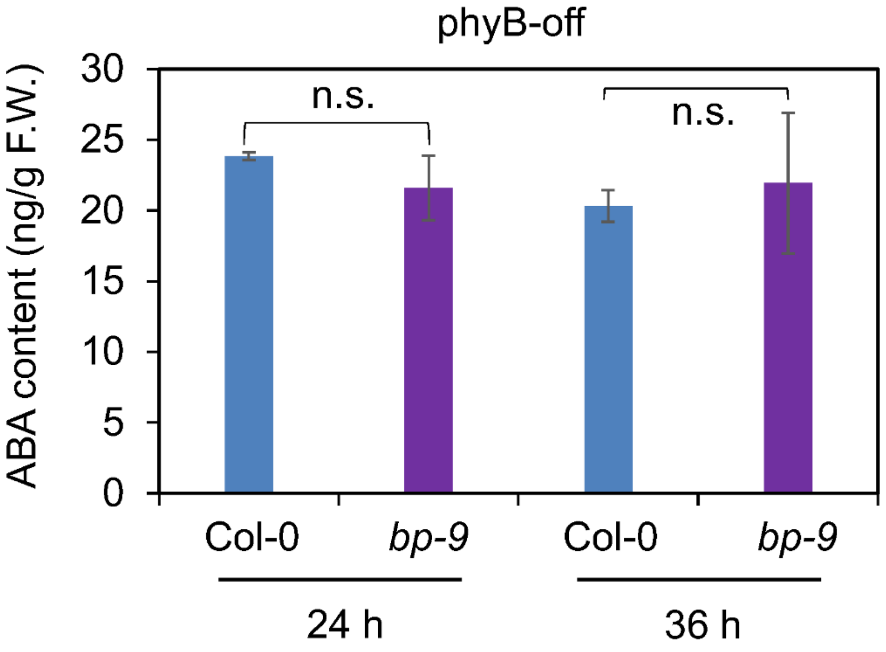

Supplemental Figure 10. Analysis of ABA levels in imbibed Col-0 and *bp-9* seeds 24 and 36 h after phyB-off treatment. Values are shown as means ± SD (Student's *t*-test, * $p < 0.05$, n=3. The n.s. means no significance.

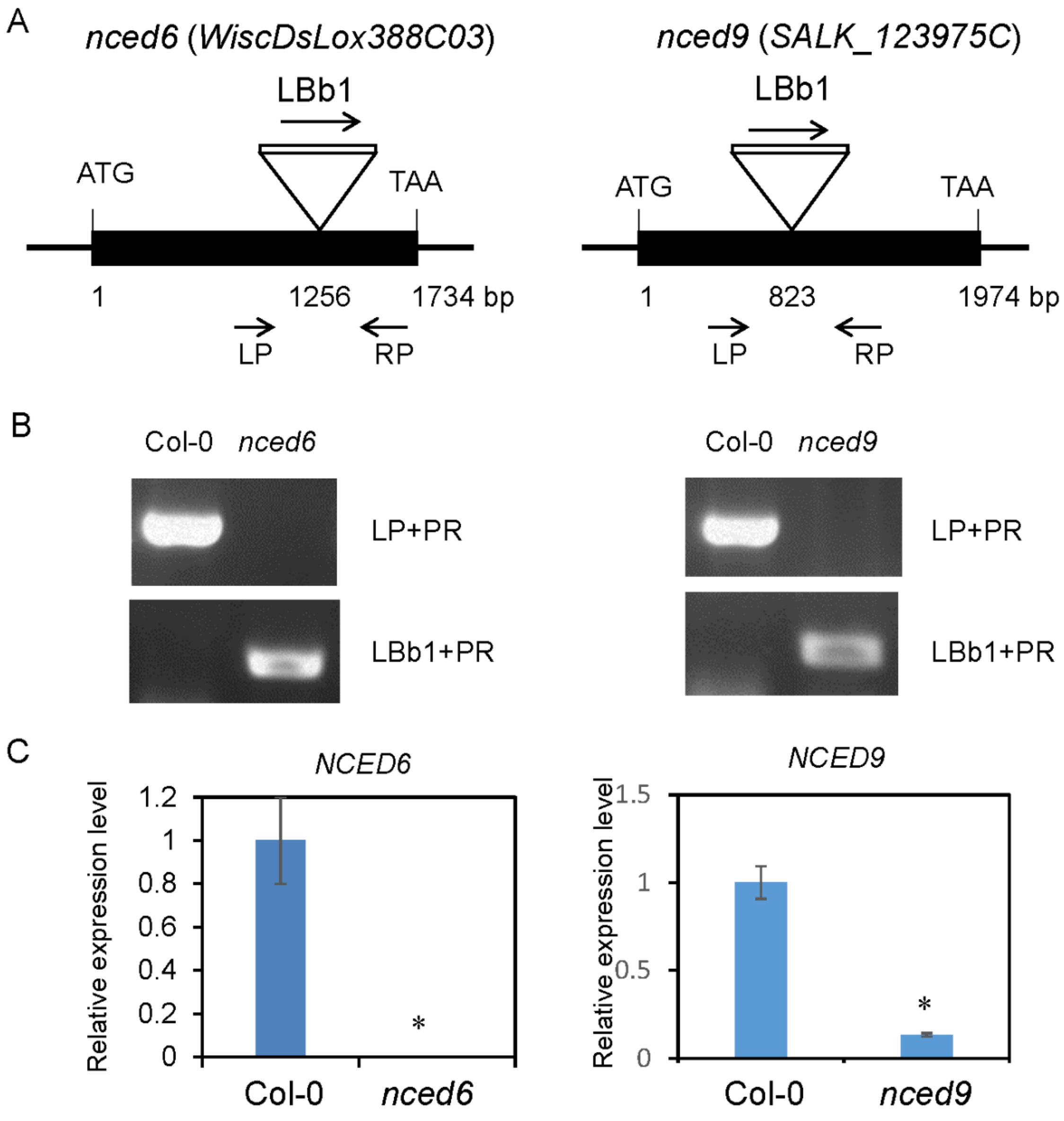

Supplemental Figure 11. Genotyping of *nced6* and *nced9* knockout mutants. (A) Schematic diagrams of the T-DNA insertion mutants of *NCED6* and *NCED9*. (B) Identification of *nced6* and *nced9* homozygous mutants by PCR analysis. (C) RT-qPCR validation of the *nced6* and *nced9* mutants. *PP2A* was used as the internal control. Values are shown as means ± SD (Student's *t*-test, * $p < 0.05$, n=3).

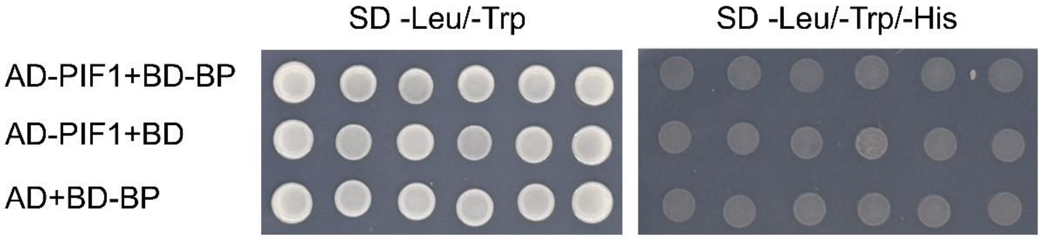

Supplemental Figure 12. Yeast two-hybrid analysis of the interaction of PIF1 with BP. PIF1 and BP fused with AD and BD vectors were co-transformed into yeast cells. The transformants were plated on SD/-Leu/-Trp and SD/-Leu/-Trp/-His dropout medium.

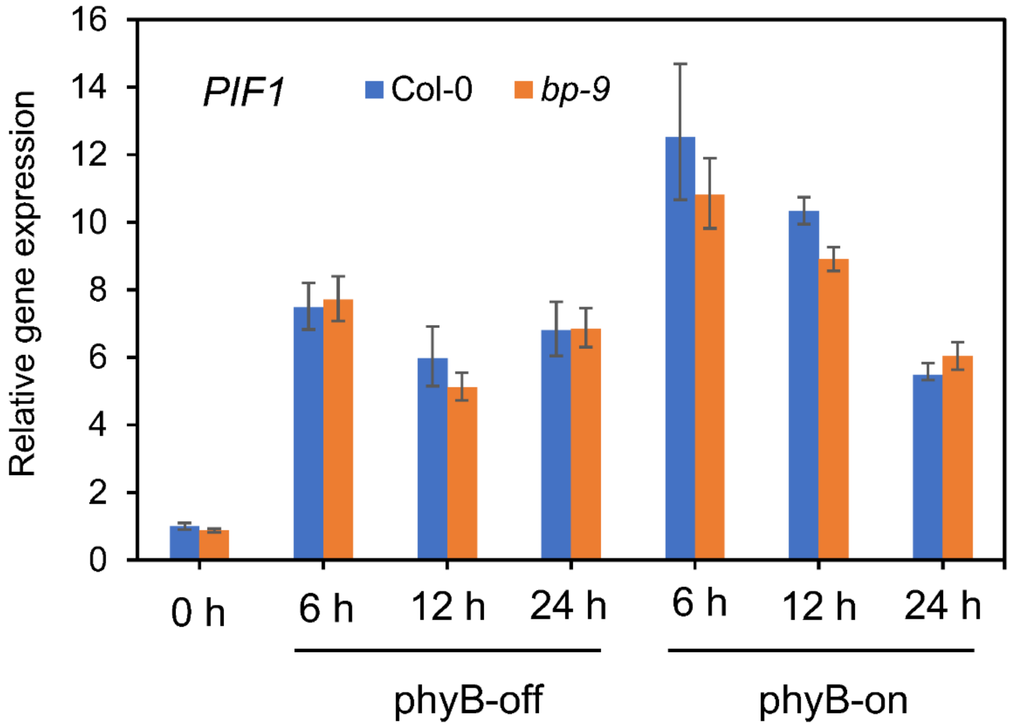

Supplemental Figure 13. RT-qPCR analysis of the expression levels of *PIF1* in imbibed Col-0 and *bp-9* seeds under phyB-on and phyB-off conditions. *PP2A* was used as internal control. Values are shown as means ± SD (Student's *t*-test, * p < 0.05, n=3).

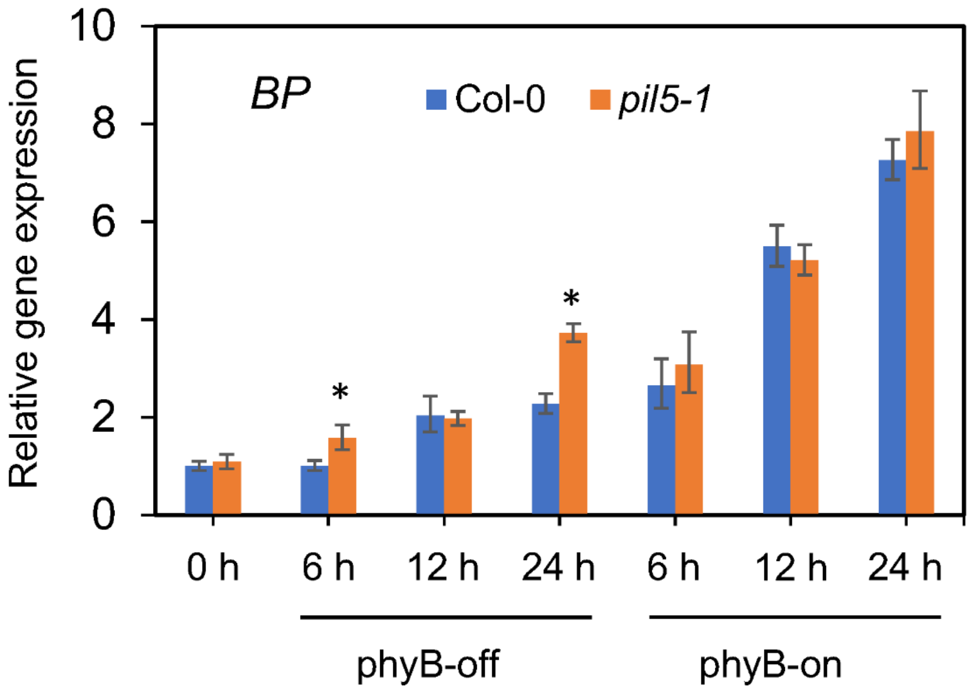

Supplemental Figure 14. RT-qPCR analysis of the expression levels of *BP* in imbibed Col-0 and *pil5-1* seeds under phyB-on and phyB-off conditions. *PP2A* was used as internal control. Values are shown as means ± SD (Student's *t*-test, * $p < 0.05$, n=3).

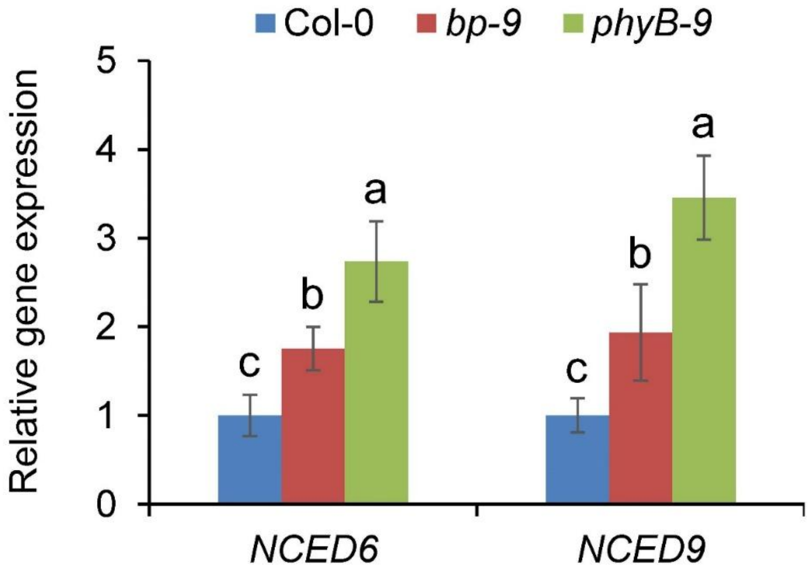

Supplemental Figure 15. RT-qPCR analysis of the expression levels of *NCED6* and *NCED9* in *bp-9* and *phyB-9* mutants in the phyB-on condition. *PP2A* was used as the internal control. Values are shown as means ± SD (n=3). The data were analyzed by one-way ANOVA followed by Tukey's HSD test. Different letters above bars indicate significant differences ($p < 0.05$).

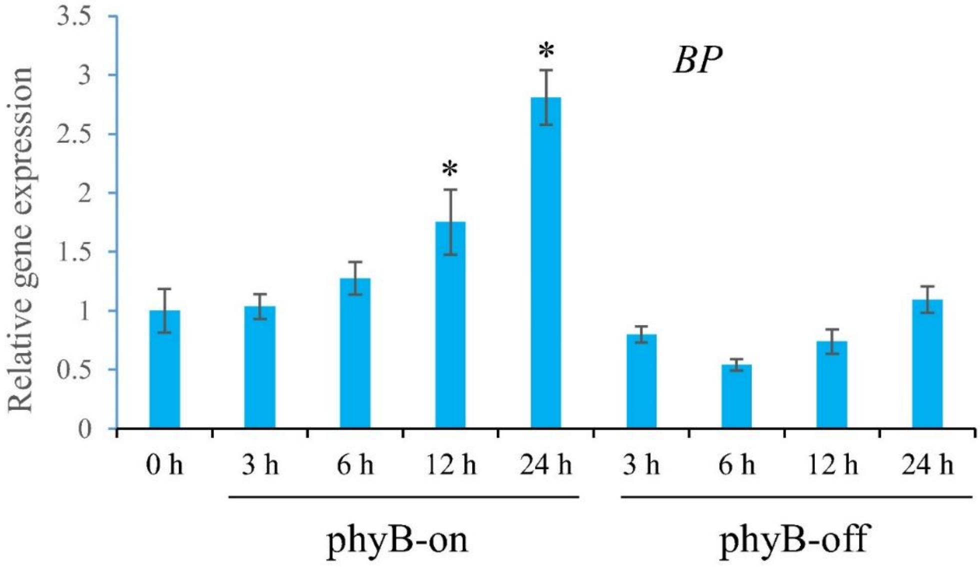

Supplemental Figure 16. RT-qPCR analysis of the expression levels of *BP* in the phyB-on and phyB-off conditions. After FR or FR/R treatments, Col-0 seeds were kept in the dark for 0, 3, 6, 12, and 24 h. *PP2A* was used the internal control. Values are shown as means ± SD (Student's *t*-test, * $p < 0.05$, n = 3).

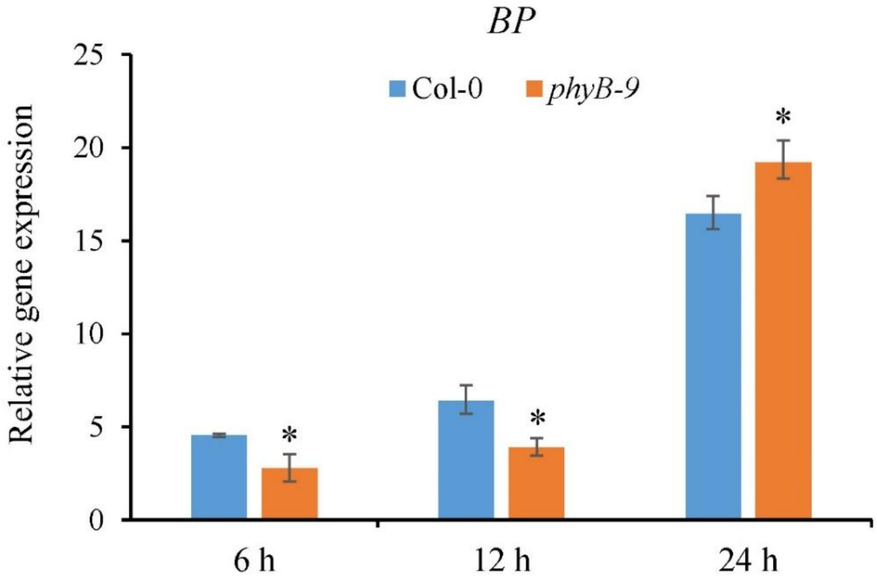

Supplemental Figure 17. RT-qPCR analysis of the expression levels of *BP* in Col-0 and the *phyB-9* mutant in the phyB-on condition. After FR/R treatments, Col-0 seeds were kept in the dark for 6, 12, and 24 h. *PP2A* was used as the internal control. Values are shown as means ± SD (Student's *t*-test, * $p < 0.05$, n = 3).

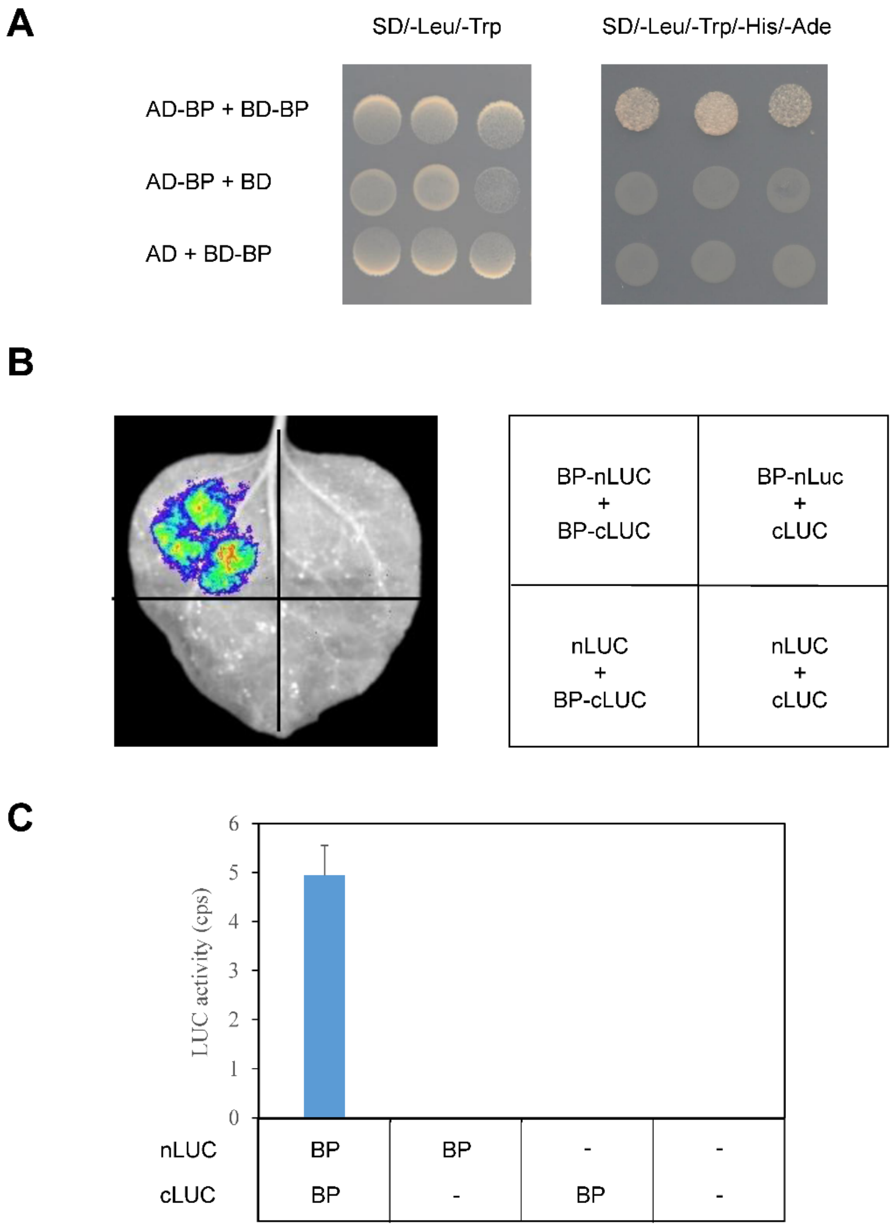

Supplemental Figure 18. BP proteins form homodimers in yeast and in plant cells. (A) Yeast two-hybrid analysis of BP-BP interaction. BP either fused with AD or BD vectors were co-transformed into yeast cells. The transformants were plated on SD/-Leu/-Trp and SD/-Leu/-Trp/-His/-Ade dropout medium. (B) LCI analysis of BP-BP interaction. BP either fused with nLUC or cLUC vectors were co-transformed into tobacco leaves and LUC luminescence signal was detected. (C) Quantification of relative LUC levels (counts per second, cps) as shown in (B). Values are shown as means ± SD.

Supplemental Table 1. LC-MS/MS analysis of the ubiquitination sites in BP protein.

Protein names	MW [kDa]	Protein score	Sequence coverage (%)	Position	Peptide score	Modified sequence	Mass error [ppm]	# PSMs
BP	45.81	14304.58	71.11	K281	22.22	AEDRELkNHLLK	0.08	1
				K135	21.31	AIHNTQEANNNNNDNVSDVEAMkAk	-3.08	1
				K137	58.83	AkIIAHPHYSTLLQAYLDcQK	1.75	1
				K207	64.82	DPELDQFMEAYcDMLVkYR	2.60	1
				K361	23.4	HWkPSEDMQFMVMDGLQHPHHAALYMDGHYMGDGPYR	2.55	1
				K156	46.63	IIAHPHYSTLLQAYLDcQkIGAPPDVVDR	0.64	1
				K287	32.01	kYSGYLSSLK	-2.94	1
				K325	45.17	LLTWWELHYkWPYPSESEK	2.56	2
				K315	29.19	QkLLTWWELHYK	-0.42	2
				K334	141.34	WPYPSESEkVALAESTGLDQK	1.11	2
				K296	64.38	YSGYLSSLkQELSK	-1.23	2

Supplement Table 2. Primers used in this study.

Primer	Sequence (5'-3')
**Mutant screening**	
LBb1	ATTTTGCCGATTTCGGAAC
nced6-LP	TTTTGAAATGCATATGAGAGTGTG
nced6-RP	CTCTTCAGTGATAGCGCATCC
nced9-LP	GCAGCGTTTAATCAAGAATCG
nced9-RP	ATCATCGTTGGTTGATCAAGC
**Construction**	
BD-BP-pr1	GGAGGCCAGTGAATTCATGGAAGAATACCAGCATGACAAC
BD-BP-pr2	CACCCGGGTGGAATTCTGGACCGAGACGATAAGGTC
AD-PHYB-N-pr1	GCCATGGAGGCCAGTGAATTCATGGTTTCCGGAGTCGGG
AD-PHYB-N-pr2	ATGCCCACCCGGGTGGAATTCACCTAACTCATCAAT
AD-PHYB-C-pr1	GCCATGGAGGCCAGTGAATTCGAAACTGCGGAAATGGATGCG
AD-PHYB-C-pr2	ATGCCCACCCGGGTGGAATTCATATGGCATCATCAG
**qRT-PCR**	
PP2A-RT-pr1	GTGACTTGGTTGAGCATTTCACTCC
PP2A-RT-pr2	GAGCTGATTCAATTGTAGCAGCAAACT
NCED6-RT-pr1	GGTCGGATATAAATTGGGTTG
NCED6-RT-pr2	CGGGTTGGTTCTCCTGATTC
NCED9-RT-pr1	AACCGCCGCTATGGTTTTAGACG
NCED9-RT-pr2	CCAGTCACCGGAAGGTTATGCAC
ABA1-RT-pr1	GATGCAGCCAAATATGGGTCAAGG
ABA1-RT-pr2	GCCATTGCATGGATAATAGCGACTC
ABA2-RT-pr1	AGAGGTGTTTGCATGATTCCTGAGC
ABA2-RT-pr2	TCCAGTGATCAATGCCACTTTACCC
ABI3-RT pr1	CTTGAAGCAAAGCGACGTGG
ABI3-RT-pr2	TGTCTTACTTTAACCCCTCGTAT
ABI4-RT-pr1	TCCGCTCAACGCAAACG
ABI4-RT-pr2	TTGTCGAACGCCACGGTA
ABI5-RT-pr1	CAATAAGAGAGGGATAGCGAACGAG
ABI5-RT-pr2	CGTCCATTGCTGTCTCCTCCA
BP-RT-pr1	GGGAAGAGTGACAATATGGG
BP-RT-pr2	TATGGACCGAGACGATAAGG
**ChIP-qPCR**	
TA3-ChIP-F	GATTCTTACTGTAAAGAACATGGCATTGAGAGA
TA3-ChIP-R	TCCAAATTTCCTGAGGTGCTTGTAACC
ACTIN2-ChIP-F	GCACCCTGTTCTTCTTACCG
ACTIN2-ChIP-R	AACCCTCGTAGATTGGCACA

NCED6-P1-F	GATTCGACACCTATTTAGGTTC
NCED6-P1-R	ATGTTCTCTACTATAATGTGGAGT
NCED6-P2-F	GAAGTGGTTGGTCAGATTCC
NCED6-P2-R	TACCGGCAGCTGTAACTAAC
NCED6-P3-F	TCGGACGGTTCGGATTCG
NCED6-P3-R	TCATGAATCATCGTTGGTTCAGG
NCED9-P1-F	AAGCAAGCGCGTAAGACATAC
NCED9-P1-R	CTGACGAAGAATACACACAACAC
NCED9-P2-F	CGCAGCGTTTAATCAAGAATCG
NCED9-P2-R	TGAAGTTGAGAAAGTTCGGTCG
NCED9-P3-F	ACGGAGGAGAGCCTCTGTTTC
NCED9-P3-R	CGGCGTTTATGAGTTGGAGTTCC
**EMSA analysis**	
NCED6-F-biotin	TAGCCGGACATCATTTATTTGACGGTGACGGAATGATTCACGCCGTTA
NCED6-R-biotin	TAACGGCGTGAATCATTCCGTCACCGTCAAATAAATGATGTCCGGCTA
NCED6-M-F	TAGCCGGACATCATTTATTAAAAGGAAAAGGAATGATTCACGCCGTTA
NCED6-M-R	TAACGGCGTGAATCATTCCTTTTCCTTTTAATAAATGATGTCCGGCTA
NCED6-M-F-biotin	TAGCCGGACATCATTTATTAAAAGGAAAAGGAATGATTCACGCCGTTA
NCED6-M-R-biotin	TAACGGCGTGAATCATTCCTTTTCCTTTTAATAAATGATGTCCGGCTA
NCED9-F-biotin	CATTACACTTTTTTAAAATGACTGGAATGCATGACGTTTGGGCAC
NCED9-R-biotin	GTGCCCAAACGTCATGCATTCCAGTCATTTTAAAAAAGTGTAATG
NCED9-M-F	CATTACACTTTTTTAAAAAAAATGGAATGCAAAAAGTTTGGGCAC
NCED9-M-R	GTGCCCAAACTTTTTGCATTCCATTTTTTTTAAAAAAGTGTAATG
NCED9-M-F-biotin	CATTACACTTTTTTAAAAAAAATGGAATGCAAAAAGTTTGGGCAC
NCED9-M-R-biotin	GTGCCCAAACTTTTTGCATTCCATTTTTTTTAAAAAAGTGTAATG
NCED6-F-cold	TAGCCGGACATCATTTATTTGACGGTGACGGAATGATTCACGCCGTTA
NCED6-R-cold	TAACGGCGTGAATCATTCCGTCACCGTCAAATAAATGATGTCCGGCTA
NCED9-F-cold	CATTACACTTTTTTAAAATGACTGGAATGCATGACGTTTGGGCAC
NCED9-R-cold	GTGCCCAAACGTCATGCATTCCAGTCATTTTAAAAAAGTGTAATG
**Dual-luciferase reporter analysis**	
SK-BP-F	TAGAACTAGTGGAT ATGGAAGAATACCAGCATGACA
SK-BP-R	CGGTATCGATAAGCTTTTATGGACCGAGACGATAAGGT
NCED9pro-F	TATAGGGCGAATTGGGTACCGTGAAATTACTAAGTACTGTAGCAAGTGTA
NCED9pro-R	TTGGCGTCTTCCATGGTCACGCTACTATTTTCTCATCTTTTAACAC
NCED6extron-F	TATAGGGCGAATTGGGTACCTCGAAATTCTGTATTCTGAAAACATTCGAC
NCED6extron-R	TTGGCGTCTTCCATGGTTGAACAAGCCGGTTTGTTTTAGTGTACCG
