# Supplementary material for: Phytochrome B stabilizes the KNOX transcription factor BP/KNAT1 to promote light-initiated seed germination in Arabidopsis thaliana
Source: Plant Commun. 2025 Sep 4;6(11):101517. doi: 10.1016/j.xplc.2025.101517 (PMC12785155; doi:10.1016/j.xplc.2025.101517)
Supplement: Document S2. Article plus supplemental information [file mmc3.pdf]

Plant Communications
Research article

CellPress
Partner Journal

Phytochrome B stabilizes the KNOX transcription factor BP/KNAT1 to promote light-initiated seed germination in *Arabidopsis thaliana*

Dachuan Gu[1,2,5], Yahan Wang[1,2,5], Minglei Zhao[3,5], Hangcong Chen[1,2], Shuhua Wu[4], Xia Jin[1,2], Ling Deng[1,2], Rujun Ji[1,2], Jingyan Xu[1,2], Feng Zheng[1] and Xuncheng Liu[1,2,*]

[1]Guangdong Provincial Key Laboratory of Applied Botany, South China Botanical Garden, Chinese Academy of Sciences, Guangzhou 510650, China

[2]College of Life Sciences, University of Chinese Academy of Sciences, Beijing 100049, China

[3]College of Horticulture, South China Agricultural University, Guangzhou, China

[4]Guangdong Provincial Key Laboratory of Tea Plant Resources Innovation and Utilization, Tea Research Institute, Guangdong Academy of Agricultural Sciences, Guangzhou 510640, China

[5]These authors contributed equally to this article.

***Correspondence: Xuncheng Liu (xunchengliu@scbg.ac.cn)**

https://doi.org/10.1016/j.xplc.2025.101517

ABSTRACT

Seed germination represents a critical step in the life cycle of plants. The far-red/red light photoreceptor phytochrome B (phyB) plays a dominant role in promoting germination by modulating gibberellin (GA) and abscisic acid (ABA) metabolism, although the underlying mechanism remains poorly understood. In this study, we identified BREVIPEDICELLUS (BP)/KNAT1, a KNOX transcription factor that acts downstream of phyB and activates light-initiated seed germination in *Arabidopsis thaliana*. BP undergoes ubiquitination and is degraded through the 26S proteasome pathway. phyB directly interacts with BP and stabilizes BP protein by decreasing its ubiquitination in imbibed seeds. A genome-wide transcriptomic analysis revealed that BP and phyB co-regulate the expression of genes associated with ABA biosynthesis, signal transduction, seed dormancy, and cell wall organization. BP suppresses the expression of two key ABA biosynthetic genes, *NCED6* and *NCED9*, thus lowering ABA levels in imbibed seeds under phyB-activated conditions. BP directly binds to *NCED6* and *NCED9* and represses their expression by increasing the levels of H3K27me3, a repressive histone modification marker. Genetic analysis demonstrated that *NCED6*/*NCED9* acts epistatic to *BP* in phyB-dependent germination. Overall, this study reveals a transcriptional module consisting of phyB-BP-*NCED6/9*, which transfers light signals that inhibit ABA biosynthesis, thereby promoting light-induced germination.

Key words: H3K27me3, KNOX, light-regulated seed germination, *NCED* genes, phyB

Gu D., Wang Y., Zhao M., Chen H., Wu S., Jin X., Deng L., Ji R., Xu J., Zheng F., and Liu X. (2025). Phytochrome B stabilizes the KNOX transcription factor BP/KNAT1 to promote light-initiated seed germination in *Arabidopsis thaliana*. Plant Comm. **6**, 101517.

INTRODUCTION

Seed germination is the first step in the growth of flowering plants, and its precise regulation is essential to ensure favorable conditions for seed development (Finch-Savage and Leubner-Metzger, 2006). Among various environmental cues, light is a major factor influencing germination. It is perceived by multiple types of photoreceptors, including phytochromes, cryptochromes, phototropins, and UVR8 (Kami et al., 2010; Li et al., 2011). Phytochromes are red/far-red (R/FR) light receptors that play a crucial role in promoting seed germination (Shinomura et al., 1994). They are synthesized in the cytosol in an inactive form, Pr, which is converted into the biologically active form, Pfr, upon red-light irradiation. *Arabidopsis thaliana* possesses five phytochrome family members, designated phyA–phyE. Among these, phyB plays a dominant role in promoting seed germination, whereas phyA functions only as a subordinate regulator in the absence of phyB (Shinomura et al., 1994, 1996).

Phytochrome-mediated seed germination mainly relies on the integration of light signaling with abscisic acid (ABA) and gibberellin (GA) pathways (Carrera-Castano et al., 2020; Li et al., 2022a). ABA and GA exhibit antagonistic effects on germination control: ABA suppresses, while GA promotes, the completion of

 © 2025 The Author(s). Published by Elsevier Inc. on behalf of CAS Center for Excellence in Molecular Plant Sciences, Chinese Academy of Sciences, and Chinese Society for Plant Biology. This is an open access article under the CC BY license (http://creativecommons.org/licenses/by/4.0/).

germination (Finch-Savage and Leubner-Metzger, 2006; Seo et al., 2009). The level of endogenous ABA is regulated by the balance between its biosynthesis and catabolism. The major ABA biosynthetic pathway is regulated by multiple factors, including the rate-limiting 9-cis-epoxycarotenoid dioxygenases (NCEDs), zeaxanthin epoxidase (ZEP/ABA1), the short-chain dehydrogenase ABA2, molybdenum cofactor sulfurase ABA3, and aldehyde oxidase AAO3 (Seo and Koshiba, 2002). NCED6 and NCED9 are key enzymes that regulate ABA biosynthesis in developing seeds (Lefebvre et al., 2006). Consistent with these roles, the *nced5 nced6 nced9* loss-of-function triple mutant germinates more rapidly than the wild type (Frey et al., 2012), whereas *NCED6*-overexpressing lines display reduced germination (Martinez-Andujar et al., 2011). Red light reduces ABA levels in imbibed seeds by repressing the transcription of ABA anabolic genes (*ABA1*, *NCED6*, and *NCED9*) and activating that of the ABA catabolic gene *CYP707A2* (Toyomasu et al., 1998; Seo et al., 2006; Oh et al., 2007; Kim et al., 2008; Yang et al., 2020).

Recent studies have identified multiple factors that modulate light-dependent seed germination by regulating ABA synthesis. Phytochrome-Interacting Factor 1 (PIF1), a master repressor of light-regulated germination, activates the transcription of *ABA1, NCED6,* and *NCED9* through its downstream transcription factor SOMNUS (SOM) (Kim et al., 2008). The AP2/ERF transcription factors ERF55 and ERF58 indirectly suppress the expression of ABA biosynthetic genes by promoting the transcription of *PIF1* and *SOM* in imbibed seeds (Li et al., 2022b). The noncoding RNA *HIDDEN TREASURE 1* (*HID1*) inhibits *NCED9* expression by reducing histone H3 trimethylation at lysine 4 (H3K4me3) (Wang et al., 2023a). However, the regulatory mechanism linking light, phyB, and ABA metabolism in modulating seed germination remains poorly defined.

In *Arabidopsis*, the KNOTTED1-like HOMEOBOX (KNOX) transcription factor BREVIPEDICELLUS (BP, also known as KNAT1) is essential for multiple developmental processes, including maintenance of the shoot meristem (Byrne et al., 2002; Smith et al., 2002), differentiation of secondary xylem (Woerlen et al., 2017), and inflorescence architecture (Ragni et al., 2008; Zhao et al., 2015). BP is associated with the SWI2/SNF2 chromatin-remodeling ATPase BRAHMA (BRM) during the regulation of inflorescence architecture; in conjunction with BRM, BP represses the downstream genes *KNAT2* and *KNAT6* by decreasing H3K4me3 levels (Zhao et al., 2015). However, the involvement of BP in seed germination, particularly in response to light, has not been characterized.

In this study, we demonstrated that BP plays a positive role in light-induced seed germination. It is ubiquitinated and degraded via the 26S proteasome. phyB interacts directly with BP and enhances its accumulation by reducing ubiquitination in imbibed seeds. Genome-wide transcriptomic analysis revealed that BP is a crucial component in phyB-mediated transcriptional reprogramming in imbibed seeds. It directly suppresses *NCED6* and *NCED9* expression by increasing H3K27me3 modification. Collectively, these findings identify the phyB-BP-*NCED6/9* module as a novel light-responsive transcriptional cascade that inhibits ABA biosynthesis and promotes seed germination.

RESULTS

BP is a positive regulator of phyB-dependent seed germination

Previous studies have demonstrated that phyB plays a major role in light-initiated seed germination (Shinomura et al., 1994, 1996). To determine whether BP participates in phyB-dependent germination, we first examined the germination rates of *BP* mutants using a phyB-dependent seed germination assay. After sterilization and imbibition under white light (WL) for 1 h, seeds were irradiated with either far-red light (phyB-off, designated FR) or far-red followed by red light (phyB-on, designated FR/R). Germination rates were recorded at multiple time points after dark treatment (Figure 1A).

Under phyB-on conditions, the germination rate of *bp-9*, a null mutant of *BP* (Douglas et al., 2002), was significantly reduced compared with the wild type Col-0 (Figures 1B and 1C). To confirm the role of BP in phyB-dependent germination, we generated transgenic lines expressing *BP* under its native promoter in the *bp-9* background. Seeds from two stable transgenic lines, *BP-GFP-1* and *BP-GFP-2*, showed germination rates comparable to Col-0 (Figures 1B and 1C). Under phyB-off conditions, neither *bp-9* seeds nor *BP-GFP* transgenic seeds germinated 4 d after FR irradiation (Supplemental Figures 1A and 1B). In contrast, all tested seeds germinated well under WL (Figures 1D and 1E). To rule out the possibility that reduced germination of *bp-9* was caused by altered dormancy, we assessed *bp-9* seed germination with and without stratification at 4°C. In both cases, *bp-9* seeds exhibited extremely high germination rates without significant differences compared to Col-0 (Supplemental Figure 2). These results suggest that the reduced germination of *bp-9* is independent of seed dormancy.

We next examined the time course of germination rates in *bp-9*, *BP-GFP*, and the *BP* overexpression line *35S:BP-GUS* (Ori et al., 2000) under phyB-on conditions. *bp-9* seeds displayed significantly lower germination rates, while *35S:BP-GUS* seeds exhibited significantly higher germination rates than Col-0 at 48, 60, and 72 h after FR/R treatment (Figure 1F). *BP-GFP-1* and *BP-GFP-2* seeds demonstrated germination rates comparable to Col-0 (Figure 1F). In addition, *bp-1*, another *BP* null mutant in the Ler background (Douglas et al., 2002), showed lower germination rates compared to Ler seeds at 48, 60, and 72 h after FR/R treatment (Figure 1G). Collectively, these data confirm that BP plays a positive role in phyB-mediated seed germination in *Arabidopsis*.

Given the importance of BP in light-regulated germination, we next analyzed its transcript levels across multiple tissues of 35-day-old plants, including roots, stems, leaves, flowers, and siliques. Quantitative reverse-transcription polymerase chain reaction (RT-qPCR) revealed that *BP* expression was strongest in roots and stems, moderate in flowers and siliques, and undetectable in leaves (Supplemental Figure 3). This tissue-specific expression pattern suggests that BP functions not only in seed germination but also in root and stem development.

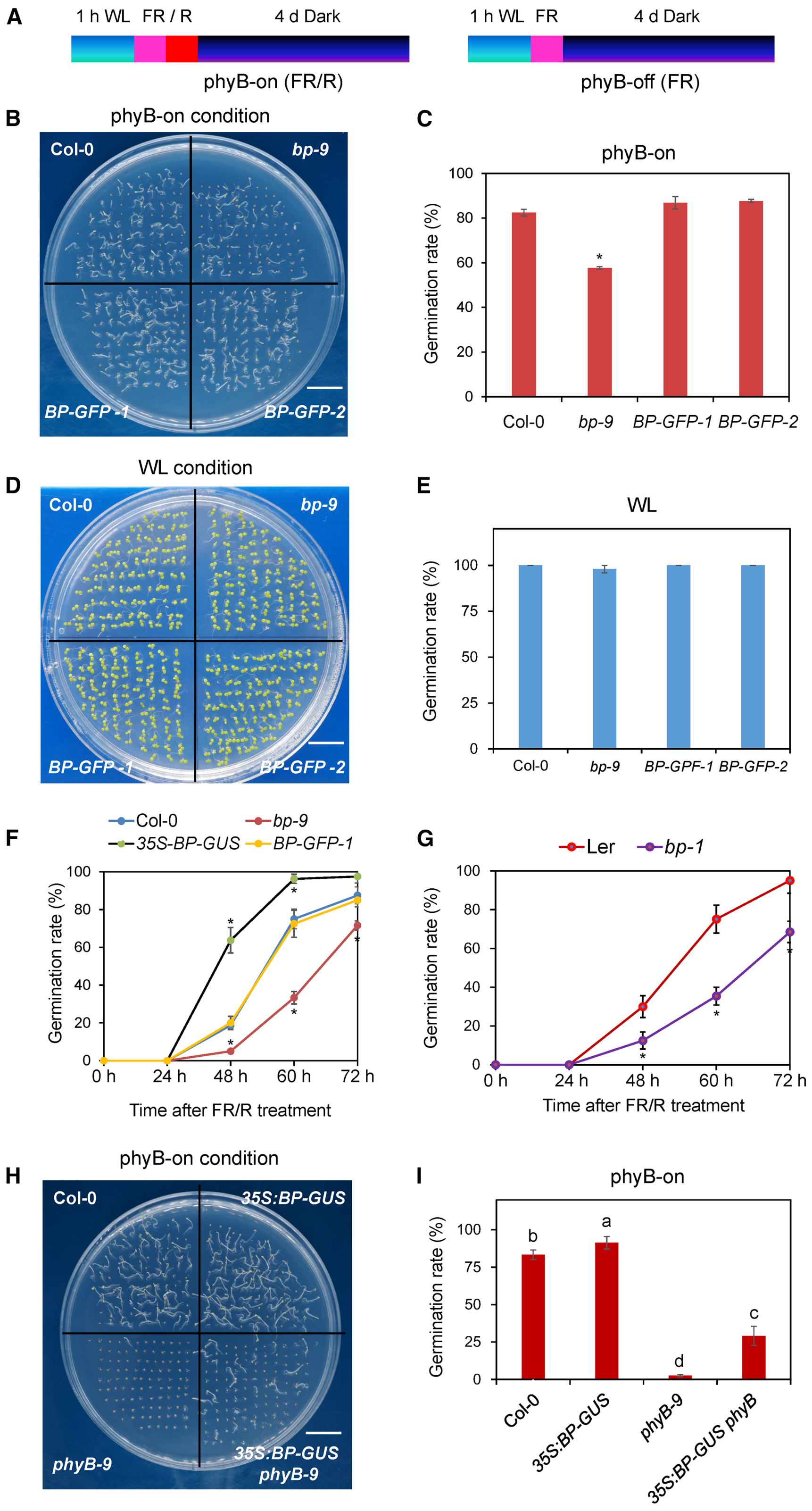

Figure 1. BP functions as a positive regulator of phyB-mediated seed germination.

(A) Schematic of the phyB-dependent seed germination assay. FR/R (phyB-on) included 5 min far-red light (3.8 μmol m^{-2} s^{-1}) followed by 5 min red light (13.1 μmol m^{-2} s^{-1}). FR (phyB-off) included 5 min far-red light (3.8 μmol m^{-2} s^{-1}). Imbibed seeds were first exposed to white light (WL) for 1 h, and then irradiated with FR/R or FR and incubated in darkness for 4 d.

(B and C) Germination phenotypes and germination rates of Col-0, *bp-9* mutant, and *BP* transgenic complementation lines (*BP-GFP-1* and *-2*) under phyB-on conditions.

(D and E) Germination phenotypes and germination rates of Col-0, *bp-9* mutant, and *BP* transgenic complementation lines under WL conditions.

(F) Time course of seed germination rates of Col-0, *bp-9*, the *35S:BP-GUS* overexpression line, and *BP-GFP-1* under phyB-on conditions.

(G) Time course of seed germination rates of Ler (Landsberg erecta) and *bp-1* under phyB-on conditions. *bp-1* is a *BP* knockout mutant in the Ler background.

(H) Germination phenotypes of Col-0, *35S:BP-GUS*, *phyB-9*, and *35S:BP-GUS phyB-9* seeds under phyB-on conditions.

(I) Statistical analysis of germination rates of Col-0, *35S:BP-GUS*, *phyB-9*, and *35S:BP-GUS phyB-9* seeds under phyB-on conditions.

Different letters above bars indicate significant differences ($p < 0.05$).

In **(C)**, **(E)**, **(F)**, and **(G)**, values are shown as mean ± SD (Student's *t*-test, *$p < 0.05$, $n = 3$). Germination assays were performed in triplicate, with more than 50 seeds per sample used for statistical analysis.

BP acts genetically downstream of *phyB* in light-regulated seed germination

Previous studies have shown that phyB promotes light-dependent germination through downstream transcription factors such as PIF1 and ERF55/58 (Oh et al., 2004, 2006; Li et al., 2022b). The observed reduction in germination of *BP* loss-of-function mutants suggest that BP also operates downstream of phyB. To clarify the genetic relationship between *BP* and *phyB* in light-dependent seed germination, we generated *35S:BP-GUS phyB-9* plants by crossing the *35S:BP-GUS* overexpression line (Lincoln et al., 1994) with the *phyB-9* mutant (Reed et al., 1993). Consistent with a prior report (Lincoln et al., 1994), the *35S:BP-GUS* transgenic line was validated and exhibited a significantly smaller size relative to wild type (Supplemental Figure 4). The germination rates of *35S:BP-GUS phyB-9* were then examined under both phyB-on and phyB-off conditions. As previously reported (Shinomura et al., 1994, 1996), *phyB-9* seeds germinated poorly under phyB-on conditions, whereas *BP* overexpression in the *phyB-9* background partially restored germination (Figures 1H and 1I). Under phyB-off conditions, the seeds of both *35S:BP-GUS* and *35S:BP-GUS phyB-9* failed to germinate (Supplemental Figures

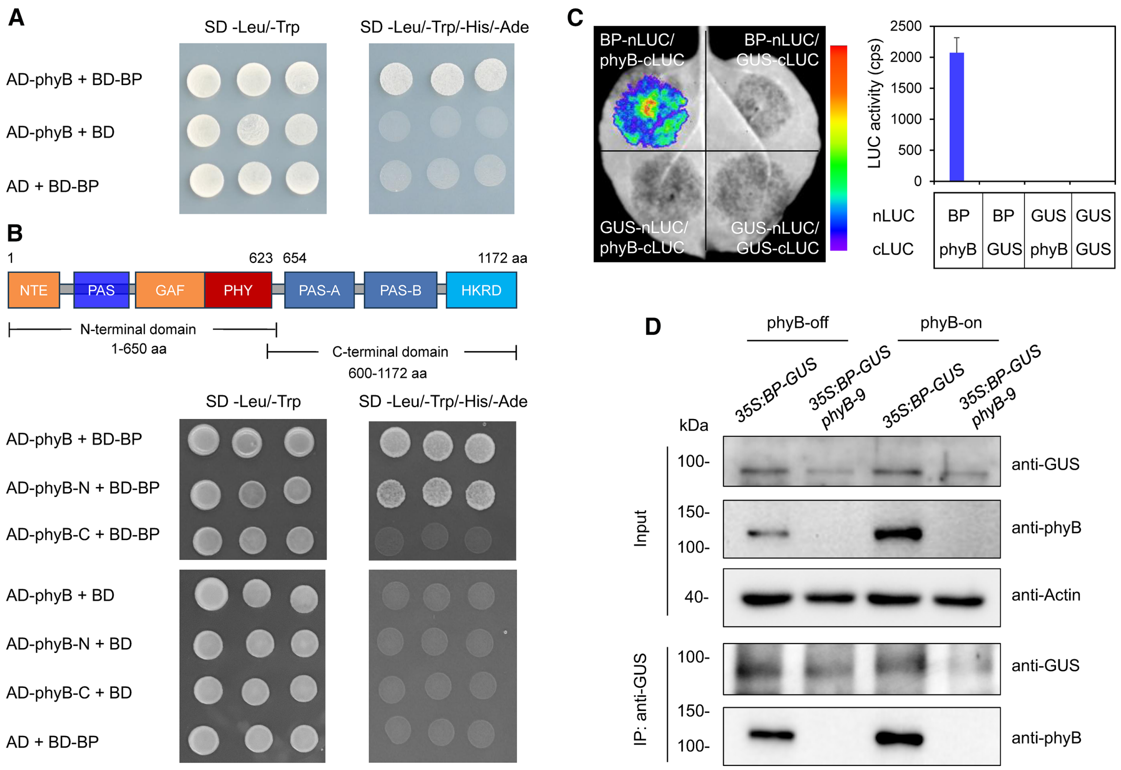

Figure 2. phyB interacts with BP *in vitro* and *in vivo*.
(A) Yeast two-hybrid analysis of the phyB–BP interaction. BP and phyB fused to AD and BD vectors were co-transformed into yeast cells. Transformants were grown on SD/−Leu/−Trp medium and SD/−Leu/−Trp/−His/−Ade dropout medium.
(B) Yeast two-hybrid analysis of phyB interaction domains with BP. The diagram illustrates N-terminal and C-terminal regions of phyB.
(C) LCI assay of the BP–phyB interaction. BP and phyB fused to nLUC and cLUC vectors were co-expressed in tobacco leaves, and luminescence signals were recorded as relative LUC activity (counts per second, cps). GUS-nLUC and GUS-cLUC served as negative controls. Values are shown as mean ± SD (n = 3).
(D) coIP assay of the BP–phyB interaction. Seeds of *35S:BP-GUS* and *35S:BP-GUS phyB-9* were treated with FR or FR/R and incubated in darkness for 24 h. Total protein was extracted, immunoprecipitated with an anti-GUS antibody, and analyzed by immunoblotting with an anti-phyB antibody.

1C and 1D). Taken together, these results suggest that *BP* functions downstream of *phyB to* promote light-dependent seed germination.

phyB interacts with BP both *in vitro* and *in vivo*

The observed genetic interaction between *BP* and *phyB* prompted us to test whether they also interact at the protein level. A yeast two-hybrid assay revealed that co-transformants of AD-phyB (phyB fused to the activation domain of GAL4) and BD-BP (BP fused to the DNA-binding domain of GAL4) grew robustly on SD/−Leu/−Trp/−His/−Ade dropout medium, indicating that they could interact in yeast cells (Figure 2A). To identify the interacting domain of phyB, the N terminus (1–650 aa) and C terminus (600–1172 aa) of phyB were fused to AD. Yeast two-hybrid assay demonstrated that the N-terminal, but not the C-terminal region of phyB interacted with BP in yeast (Figure 2B).

Next, we examined the interaction between BP and phyB using a luciferase complementation imaging (LCI) assay. Strong luciferase luminescence signals were detected in tobacco leaves co-transformed with BP-nLUC (BP fused to the N terminus of luciferase) and phyB-cLUC (phyB fused to the C terminus of luciferase) (Figure 2C), supporting *in vivo* interaction between phyB and BP.

To further validate this result, a co-immunoprecipitation (coIP) assay was performed. Seeds of *35S:BP-GUS* (transgenic lines expressing BP fused to GUS) and *35S:BP-GUS phyB-9* were treated with FR or FR/R, then incubated in darkness for 24 h. After immunoprecipitation with an anti-GUS antibody, clear phyB protein bands were detected in imbibed *35S:BP-GUS* seeds with an anti-phyB antibody under both FR and FR/R conditions (Figure 2D). Notably, the interaction between phyB and BP was stronger under phyB-on conditions than under phyB-off conditions (Figure 2D). Collectively, these results suggest that BP interacts with phyB both *in vitro* and *in vivo*.

phyB promotes the accumulation of BP by decreasing its ubiquitination

Existing evidence suggests that phytochromes initiate light responses by destabilizing their interacting proteins, such as PIFs (Al-Sady et al., 2006; Shen et al., 2007, 2008; Lorrain et al., 2008). These observations prompted us to investigate whether phyB also regulates BP protein levels in imbibed seeds. Transgenic *35S:phyB* (*35S:phyB-Flag*) lines were generated and crossed with *35S:BP-GUS* plants. *35S:BP-GUS* and *35S:BP-GUS 35S:phyB* lines showed comparable *BP* expression levels in imbibed seeds (Supplemental Figure 5). After phyB-on or phyB-off treatment, seeds of *35S:BP-GUS*,

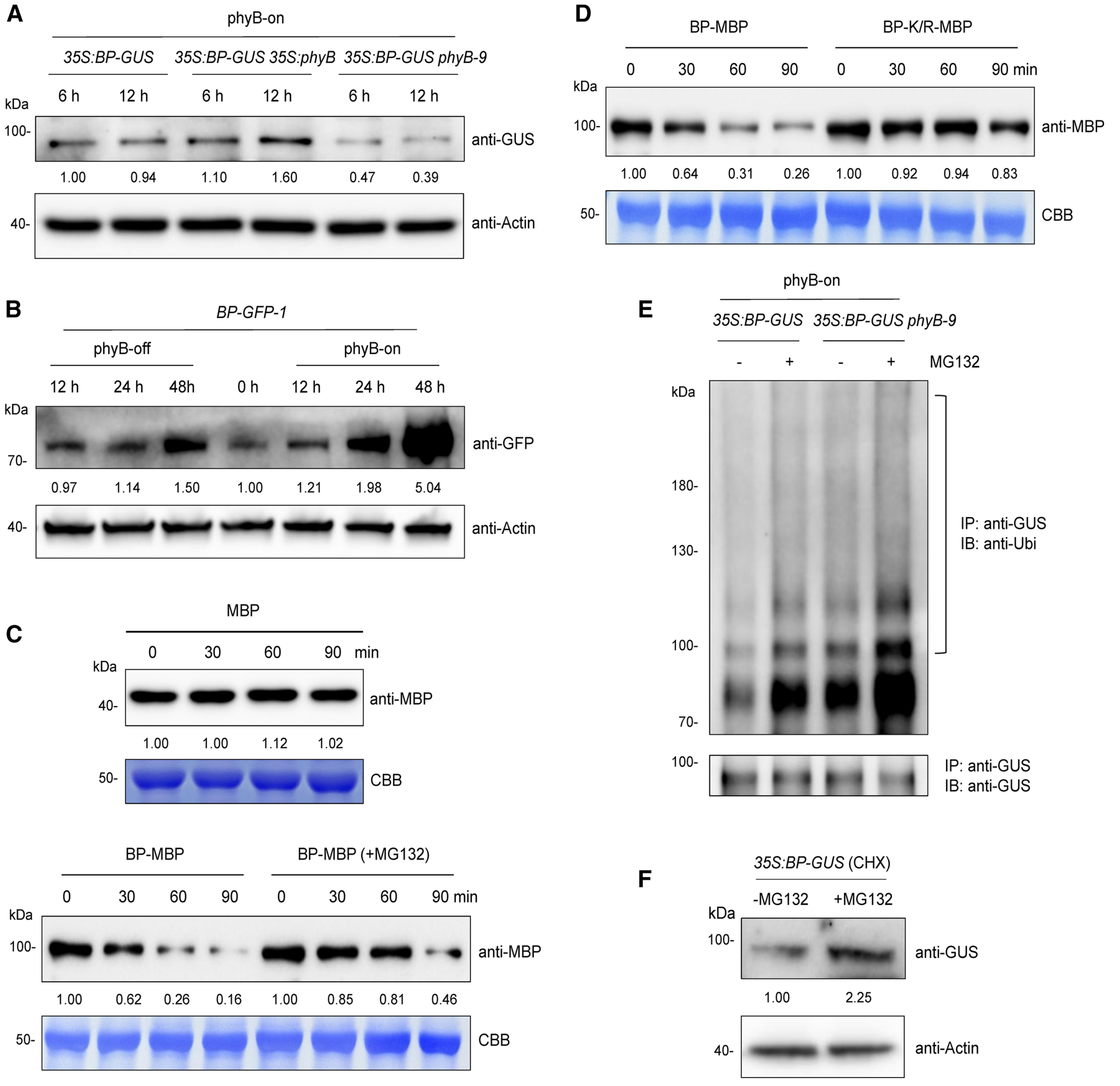

Figure 3. phyB decreases ubiquitination of BP to promote its accumulation in imbibed seeds under phyB-on conditions.

(A) Immunoblot analysis of BP protein levels in response to *phyB* overexpression and loss of function under phyB-on conditions. *35S:BP-GUS*, *35S:BP-GUS 35S:phyB*, and *35S:BP-GUS phyB-9* seeds were treated with FR/R and incubated in darkness for 6 or 12 h. Proteins were detected with an anti-GUS antibody. Relative signal intensity was normalized to the actin control.

(B) Immunoblot analysis of BP levels in imbibed *BP-GFP-1* seeds under phyB-on and phyB-off conditions. Seeds were treated with FR/R or FR and incubated in darkness for the indicated times. Relative signal intensity was normalized to the actin control.

(C) Cell-free degradation assays of BP-MBP protein incubated with crude extracts of 16-day-old Col-0 leaves for 0, 30, 60, or 90 min. Reactions included MG132 (50 μM) or no inhibitor. Relative signal intensity was normalized to Coomassie Brilliant Blue (CBB) staining. MBP protein was used as a negative control.

(D) Cell-free degradation assays of BP-MBP and BP-K/R-MBP proteins incubated with crude extracts of 16-day-old Col-0 leaves for 0, 30, 60, or 90 min. Relative signal intensity was normalized to CBB staining. K/R indicates substitution of lysine residues with arginine.

(E) Immunoprecipitation analysis of BP ubiquitination in imbibed seeds of *35S:BP-GUS* and *35S:BP-GUS phyB-9* under phyB-on conditions. Seeds were plated on 1/2 MS medium supplemented with 50 μM MG132 (or no inhibitor), treated with FR/R, and kept in the dark for 12 h. Total proteins were immunoprecipitated with an anti-GUS antibody and probed with a pan anti-ubiquitin (anti-Ubi) antibody.

(F) Immunoblot analysis of the effect of MG132 on BP protein accumulation under phyB-on conditions. Seeds of *35S:BP-GUS* were plated on 1/2 MS medium supplemented with 50 μM CHX and 50 μM MG132 (or CHX only), treated with FR/R, and incubated in darkness for 12 h. BP protein was detected with an anti-GUS antibody. The values represent relative signal intensities, which were normalized to the actin control.

35S:BP-GUS 35S:phyB, and *35S:BP-GUS phyB-9* were incubated in darkness for 6 or 12 h. Immunoblot analysis revealed that BP protein levels were higher in imbibed *35S:BP-GUS* seeds than in *35S:BP-GUS phyB-9* seeds but lower than in *35S:BP-GUS 35S:phyB* seeds under phyB-on conditions (Figure 3A). Under phyB-off conditions, BP levels slightly increased in the *35S:phyB* background but decreased in the *phyB-9* mutant (Supplemental Figure 6). These data indicate that phyB may promote the accumulation of BP protein in imbibed seeds under both phyB-on and phyB-off conditions.

The stabilization of BP by phyB led us to examine its spatial expression patterns in imbibed seeds. Immunoblotting demonstrated that BP protein levels progressively increased in

imbibed *BP-GFP-1* seeds after both phyB-on and phyB-off treatments (Figure 3B). Notably, the increase in BP levels was more pronounced when phyB was active (Figure 3B). These findings indicate that BP protein accumulation in seeds is induced by light.

Ubiquitination is a well-established post-translational modification that regulates the stability of many endogenous proteins. We therefore tested whether BP undergoes ubiquitination. BP protein was expressed in *Escherichia coli*, purified, and subjected to liquid chromatography–tandem mass spectrometry (LC–MS/MS). Eleven ubiquitinated lysine residues were identified in BP (Supplemental Table 1). The stability of BP was then assessed by cell-free degradation assays. BP-MBP protein was incubated with crude extracts of Col-0 leaves for 0, 30, 60, and 90 min; MBP served as the negative control. Immunoblot assay showed that BP-MBP protein rapidly degraded within 90 min, whereas degradation was largely suppressed by MG132, an inhibitor of the 26S proteasome (Figure 3C). Moreover, mutation of all ubiquitinated lysine residues to arginine (K-R, mimicking de-ubiquitination) reduced BP degradation compared with the control (Figure 3D). These findings suggest that BP undergoes ubiquitination and is then degraded by the 26S proteasome system.

We also assessed whether phyB influences the ubiquitination level of BP under phyB-on conditions. After FR/R treatment, *35S:BP-GUS* and *35S:BP-GUS phyB-9* seeds were treated with MG132, incubated in darkness for 12 h, and harvested for analysis. BP protein was immunoprecipitated with an anti-GUS antibody. A prominent smear band was detected by the anti-ubiquitin antibody; MG132 treatment substantially increased ubiquitinated BP in both *35S:BP-GUS* and *35S:BP-GUS phyB-9* seeds (Figure 3E), suggesting that BP undergoes ubiquitination in imbibed seeds. Notably, BP ubiquitination was higher in *35S:BP-GUS phyB-9* seeds than in *35S:BP-GUS* seeds (Figure 3E), indicating that phyB reduces BP ubiquitination under phyB-on conditions.

Finally, we examined whether MG132 treatment affects BP accumulation in imbibed seeds. Seeds of *35S:BP-GUS* were plated on half-strength MS medium supplemented with the protein synthesis inhibitor cycloheximide (CHX) and MG132 (or CHX alone), then irradiated with FR/R. MG132 treatment considerably increased BP protein levels in imbibed *35S:BP-GUS* seeds (Figure 3F). Collectively, these data suggest that phyB promotes BP accumulation by decreasing its ubiquitination and subsequent 26S proteasome-mediated degradation under phyB-on conditions.

RNA-seq analysis of the BP-regulated transcriptome under phyB-on conditions

To gain further insight into the biological function of BP during light-induced seed germination, we analyzed the BP-regulated transcriptome via RNA sequencing (RNA-seq). After 1 h of imbibition and FR/R treatment, seeds of Col-0 and *bp-9* were incubated in darkness for 24 h; they were then harvested for RNA extraction, cDNA library construction, and high-throughput sequencing (Supplemental Figure 7A). Three independent biological replicates were prepared, and differentially expressed genes were defined as those displaying at least a 1.5-fold change in expression ($p < 0.05$). Compared with the wild type, 1652 genes were upregulated and 586 genes were downregulated in the *bp-9* mutant (Supplemental Datasets 1 and 2). To identify functional categories enriched among these genes, Gene Ontology (GO) enrichment analysis was performed with Metascape (https://metascape.org) (Zhou et al., 2019). Genes upregulated in *bp-9* were predominantly associated with ABA- and stress-related pathways, including responses to oxygen levels, salt stress, heat, oxidative stress, ABA stimulus, light intensity, and cold (Supplemental Figure 7B). Conversely, down-regulated genes were mainly linked to developmental processes such as photosynthesis, carboxylic acid biosynthesis, porphyrin-containing compound metabolism, DNA replication, and wax biosynthesis (Supplemental Figure 7C). These findings indicate that BP regulates transcriptional networks related to ABA signaling, stress responses, and developmental pathways in imbibed seeds.

BP is an important component of phyB-mediated transcriptional reprogramming

A recent study reported that loss of *phyB* function resulted in the upregulation of 2557 genes and downregulation of 6203 genes in imbibed seeds 24 h after FR/R treatment (Wang et al., 2023b). We thus compared BP-regulated genes with those controlled by phyB. Notably, 73.1% of BP-regulated genes (1635 of 2238) overlapped with phyB-regulated genes (Figures 4A and 4B). Among these co-regulated genes, 1050 (64.2%) were commonly upregulated, 522 genes (31.9%) were commonly downregulated, and 63 (3.9%) were differentially regulated in *bp-9* and *phyB-9* mutants (Figures 4A and 4B; Supplemental Datasets 3–5). This overlap implies that BP functions as a key component of phyB-mediated transcriptional reprogramming in imbibed seeds.

A large subset of genes associated with ABA metabolism and signaling were co-regulated by BP and phyB. These included ABA biosynthetic genes (*ABA1*, *NCED5*, *NCED6*, and *NCED9*); core ABA signaling genes (*PYL2*, *PYL7*, *PYL13*, *ABI2*, *HAB1*, *AHG1*, *HAI1*, *HAI2*, and *HAI3*); and ABA-responsive transcription factor genes (*ABI5*, *ABF4*, *RAB18*, and *DREB2A*) (Figure 4C). Notably, most of these genes were co-upregulated in *bp-9* and *phyB-9* mutants, suggesting that BP represses ABA biosynthesis and signaling in phyB-dependent seed germination.

Genes related to seed dormancy, such as *DOG1*, *DOGL4*, *RDO5*, *DRM1*, *DRM2*, and *SOM*, were upregulated, whereas genes associated with cell wall organization—including *EXPA1*, *EXPA15*, *EXT21*, *XTH15*, *XTH16*, *XTH4*, *PME18*, *PME25*, and *PME32*—were downregulated in *bp-9* and *phyB* mutants (Figure 4C). These patterns are consistent with the positive roles of these pathways in light-dependent germination. Unexpectedly, multiple genes related to chlorophyll biosynthesis and photosynthesis were also upregulated in *bp-9* and *phyB* mutants (Figure 4C), suggesting a potential role for BP in regulating phyB-mediated photomorphogenic growth.

BP regulates the expression of ABA biosynthetic genes *NCED6* and *NCED9*

ABA is a key hormone that represses seed germination. Because transcriptomic analysis demonstrated that BP and phyB

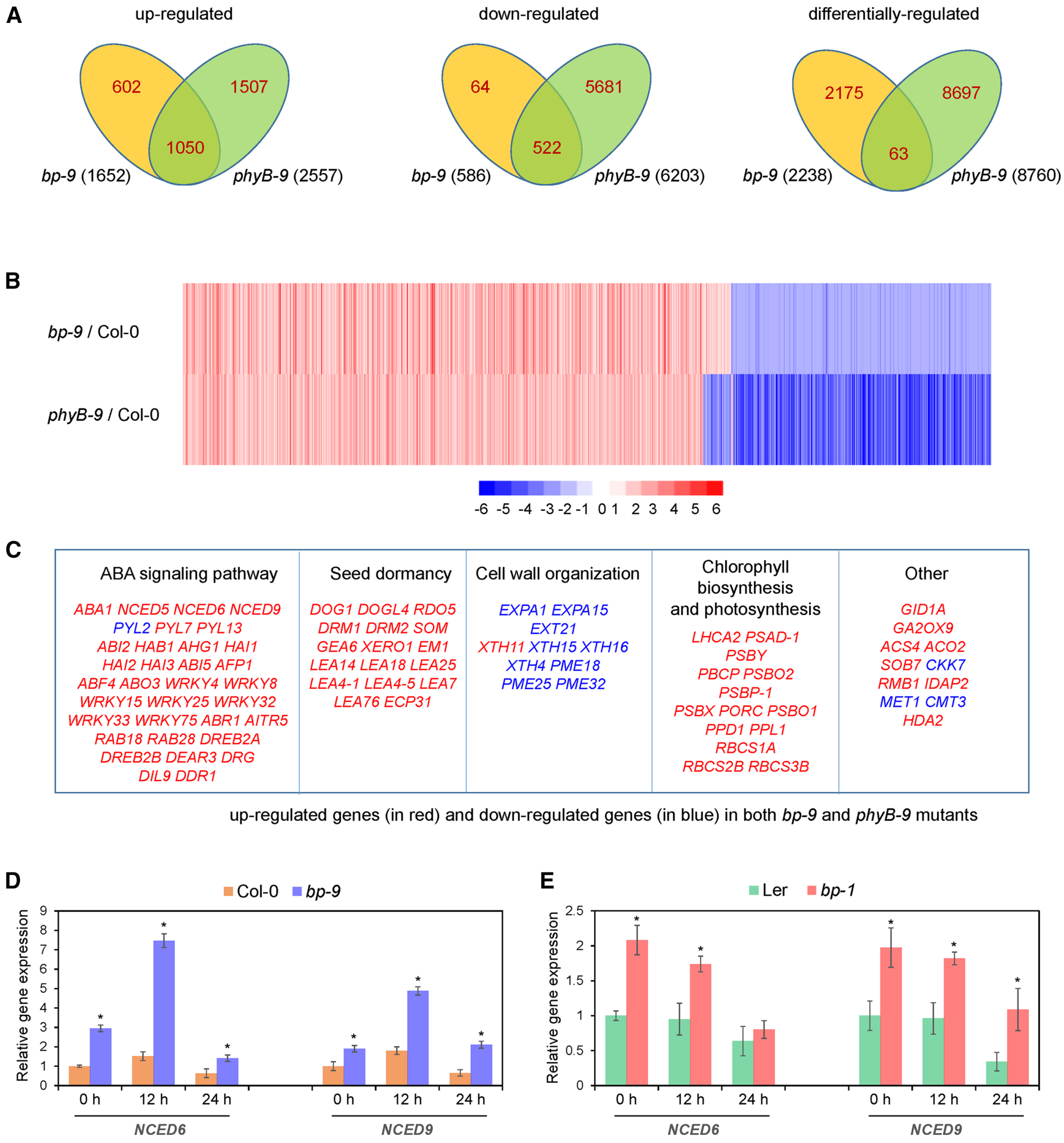

Figure 4. BP is an essential component of the phyB-regulated transcriptional network and represses *NCED6/9* expression in imbibed seeds.

(A) Venn diagram of genes co-regulated by BP and phyB 24 h after FR/R treatment.

(B) Heatmap displaying co-regulated genes in *bp-9* and *phyB-9* mutants.

(C) Key up- and downregulated genes identified in *bp-9* and *phyB-9* mutants.

(D) RT-qPCR analysis of *NCED6/9* transcript levels in Col-0 and *bp-9* seeds under phyB-on conditions.

(E) RT-qPCR analysis of *NCED6/9* transcript levels in Ler and *bp-1* seeds under phyB-on conditions.

In **(D)** and **(E)**, *PP2A* was used as an internal control. Values are shown as mean ± SD (Student's *t*-test, $*p < 0.05$, $n = 3$).

co-regulate numerous genes related to ABA biosynthesis and signaling, we investigated whether BP directly influences the expression of key ABA biosynthesis and signaling genes in imbibed seeds.

We first examined the expression patterns of these genes in *bp-9* mutants at 0, 12, and 24 h after FR/R treatment. Compared with the wild type, *bp-9* mutants showed significant increases in *NCED6* and *NCED9* transcript levels at all three time points (Figure 4D). Similar results were obtained for the *bp-1* mutant (Figure 4E). These findings confirm that BP represses the expression of the ABA biosynthetic genes *NCED6* and *NCED9* in imbibed seeds.

It has been reported that ABA inhibits seed germination through signaling components such as ABI3, ABI4, and ABI5 (Giraudat et al., 1992; Finkelstein et al., 1998; Finkelstein and Lynch, 2000). We thus examined the expression profiles of these genes in the *bp-9* mutant. Transcript levels of *ABI3*, *ABI4*, and *ABI5* were significantly upregulated in imbibed *bp-9* seeds

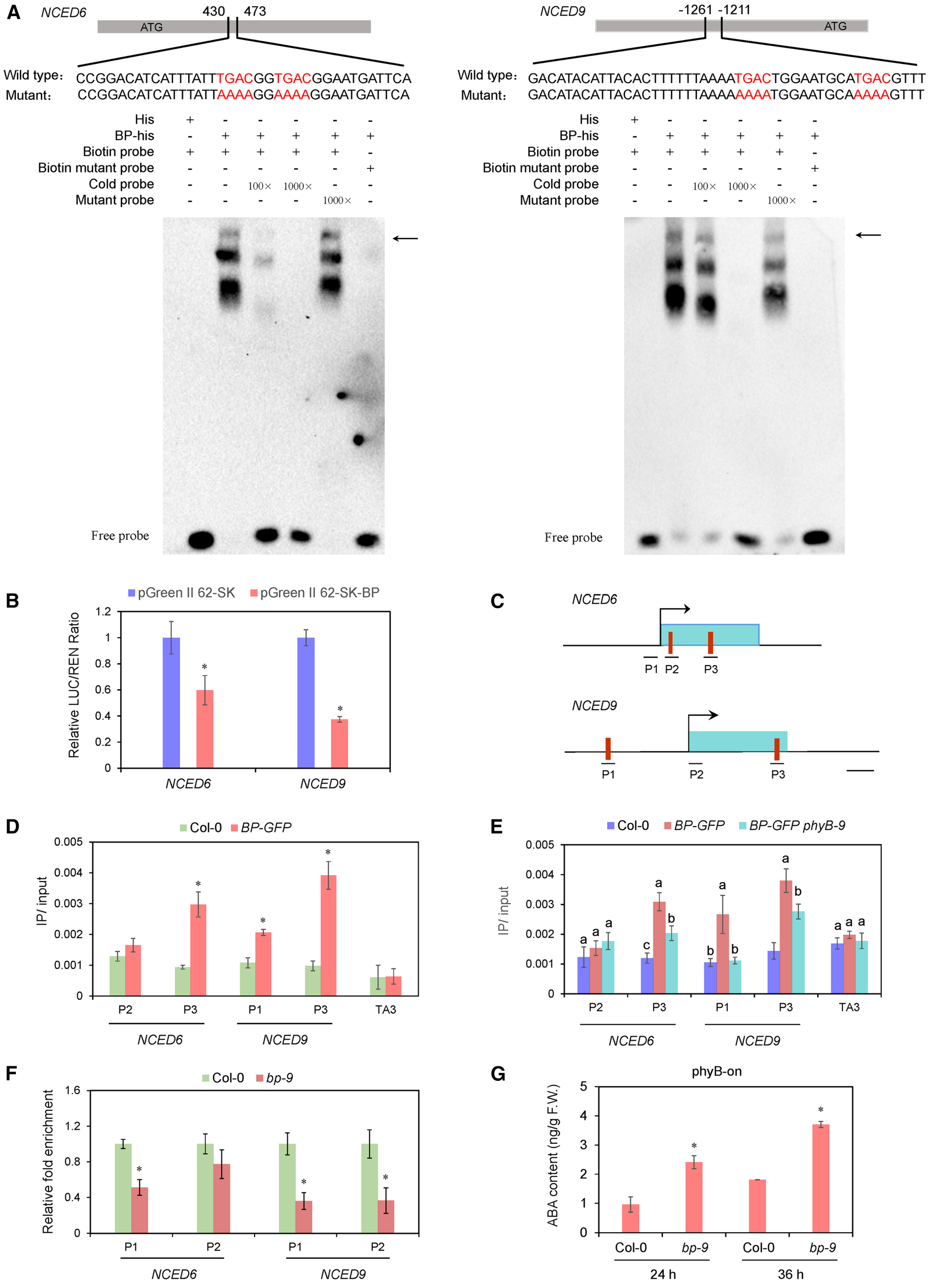

Figure 5. BP directly binds *NCED6* and *NCED9 in vitro* and *in vivo* and increases their H3K27me3 levels.

(A) EMSA analysis of BP binding to TGAC-motif-containing regions of *NCED6* (left) and *NCED9* (right). Arrows indicate shifted bands.

(B) Dual-luciferase assays demonstrating repression of *NCED6/9* by BP. Reporter and effector constructs were co-expressed in tobacco leaves via *Agrobacterium tumefaciens* GV3101. Repression is indicated by the firefly luciferase (LUC) to Renilla luciferase (REN) ratio. Values are shown as mean ± SD (Student's *t*-test, $*p < 0.05$, $n = 6$).

(C) Schematic diagrams of *NCED* genes. Red lines indicate predicted BP-binding *cis* elements (adjacent TGAC motifs). Scale bar = 0.5 kb.

(legend continued on next page)

compared with the wild type at most time points after FR/R treatment (Supplemental Figure 8). Taken together, these data suggest that BP promotes phyB-dependent germination by repressing both the ABA biosynthetic genes *NCED6* and *NCED9* and the ABA signaling genes *ABI3*, *ABI4*, and *ABI5* in imbibed seeds.

BP directly binds to *NCED6/9 in vitro* and *in vivo* and represses their expression by increasing H3K27me3 levels

To determine whether BP directly binds to *NCED6* and *NCED9 in vitro*, we performed electrophoretic mobility shift assays (EMSAs). A previous study showed that KNOX proteins target genes through a *cis*-regulatory element containing two adjacent TGAC motifs (Bolduc and Hake, 2009). BP protein fused with a His tag was expressed in *E. coli* and purified for analysis. DNA sequences containing adjacent TGAC motifs from *NCED6* and *NCED9* were synthesized for testing (Figure 5A). EMSAs showed that BP-MBP, but not MBP alone, strongly bound to the biotin-labeled probes containing TGAC motifs from *NCED6* and *NCED9*. Excess unlabeled competitor probes but not mutant probes effectively reduced this binding (Figure 5A), suggesting that BP specifically recognizes TGAC motifs in *NCED6* and *NCED9 in vitro.*

A dual-luciferase (dual-LUC) assay was then performed to evaluate whether BP represses *NCED6 and NCED9* expression. Compared with the control, the LUC/REN ratio was significantly reduced in the presence of BP (Figure 5B), indicating that BP can suppress the transcriptional activities of *NCED6* and *NCED9*.

To assess whether *NCED6* and *NCED9* are direct targets of BP *in vivo*, chromatin immunoprecipitation (ChIP) assays were performed using *BP-GFP* transgenic plants. The expression of *BP-GFP* in the *bp-9* background fully rescued the seed germination defect of *bp-9* under phyB-on conditions (Figures 1B and 1C), confirming that the BP-GFP protein is functional *in vivo*. Regions containing two adjacent TGAC motifs were selected for analysis (Figure 5C). ChIP assays showed that BP-GFP was enriched at TGAC motif-containing regions of *NCED6* and *NCED9* (Figure 5D), indicating that these genes are direct targets of BP in imbibed seeds. Additionally, we investigated whether phyB affects BP binding to *NCED6* and *NCED9*. ChIP analysis revealed that BP enrichment on *NCED6/9* was significantly reduced in *BP-GFP phyB-9* seeds compared with *BP-GFP* seeds under phyB-on conditions (Figure 5E), suggesting that phyB enhances the association of BP with *NCED6/9* in imbibed seeds. Collectively, these findings imply that BP interacts with *NCED6* and *NCED9* both *in vitro* and *in vivo*.

A previous report showed that BP interacts with the chromatin remodeling factor BRM and represses *KNAT2* and *KNAT6* expression by reducing H3K4me3 levels (Zhao et al., 2015), suggesting that BP regulates target gene expression through histone modifications. We therefore examined the levels of H3K4me3 and H3K27me3, two typical histone modifications, at *NCED6* and *NCED9* in imbibed Col-0 and *bp-9* seeds via ChIP assays. The promoter and exon regions of *NCED6* and *NCED9* were selected for analysis (Figure 5C). Levels of H3K4me3, a marker of transcriptional activation, were not significantly altered in the *bp-9* mutant compared with the wild type (Supplemental Figure 9). In contrast, levels of H3K27me3, a well-established transcriptional repression marker (Liu et al., 2010), were significantly reduced in the promoter regions of *NCED6/9* and the exon region of *NCED9* within *bp-9* seeds (Figure 5F). These results indicate that BP may repress *NCED6/9* expression by increasing levels of H3K27me3.

Direct repression of *NCED6* and *NCED9* expression by BP prompted us to measure endogenous ABA levels in imbibed wild type and *bp-9* seeds. Compared with imbibed Col-0 seeds, *bp-9* seeds accumulated significantly higher ABA levels at both 24 h and 36 h after FR/R treatment (phyB active) (Figure 5G) but showed no significant difference after FR treatment (phyB inactive) (Supplemental Figure 10). These findings confirm that BP promotes light-regulated germination by repressing ABA biosynthesis in imbibed seeds.

Genetic analysis of *BP* and *NCED6/9* in light-induced seed germination

Given that BP represses the expression of *NCED6* and *NCED9*, we investigated their genetic relationship. An earlier study indicated that *NCED6* negatively regulates phyB-dependent germination (Seo et al., 2006). Therefore, we generated *NCED6* overexpression lines and crossed them with *35S:BP-GUS* plants to analyze genetic interactions between *BP* and *NCED6*.

Three independent *35S:NCED6* lines exhibited reduced seed germination compared with Col-0 under phyB-on conditions (Figures 6A and 6B), supporting a negative role for *NCED6* in light-induced seed germination. Furthermore, overexpression of *NCED6* significantly decreased the germination rate of *35S:BP-GUS* seeds (Figures 6C and 6D), suggesting that *BP* promotes light-induced germination at least partly through repression of *NCED6*.

To more directly evaluate the genetic relationship between *BP* and *NCED6/NCED9*, we obtained loss-of-function mutants for *NCED6* and *NCED9*. Homozygous *nced6 and nced9* mutants were obtained by genotyping and validated by RT-qPCR (Supplemental Figure 11). The *nced6 nced9* double mutant and *bp nced6 nced9* triple mutant were subsequently generated by crossing. Phenotypic analysis demonstrated that *nced6 nced9* seeds displayed significantly higher germination rates than

(D) ChIP-qPCR analysis of BP enrichment at different regions of *NCED6/9* under phyB-on conditions. *BP-GFP* indicates a transgenic line expressing BP under its native promoter. An anti-GFP antibody was used for immunoprecipitation. *TA3* served as the negative control.

(E) ChIP-qPCR analysis of BP enrichment at different regions of *NCED*6/9 in *BP-GFP* and *BP-GFP phyB-9* seeds under phyB-on conditions. Values are shown as mean ± SD ($n = 3$). Data were analyzed by one-way ANOVA, followed by Tukey's HSD test. Different letters above bars indicate significant differences ($p < 0.05$).

(F) ChIP-qPCR analysis of H3K27me3 levels at *NCED6/9* in imbibed Col-0 and *bp-9* seeds. *ACTIN2* was used as an internal control. Values are shown as mean ± SD (Student's *t*-test, $^*p < 0.05$, $n = 3$).

(G) ABA content in imbibed Col-0 and *bp-9* seeds under phyB-on conditions. Values are shown as mean ± SD (Student's *t*-test, $^*p < 0.05$, $n = 3$).

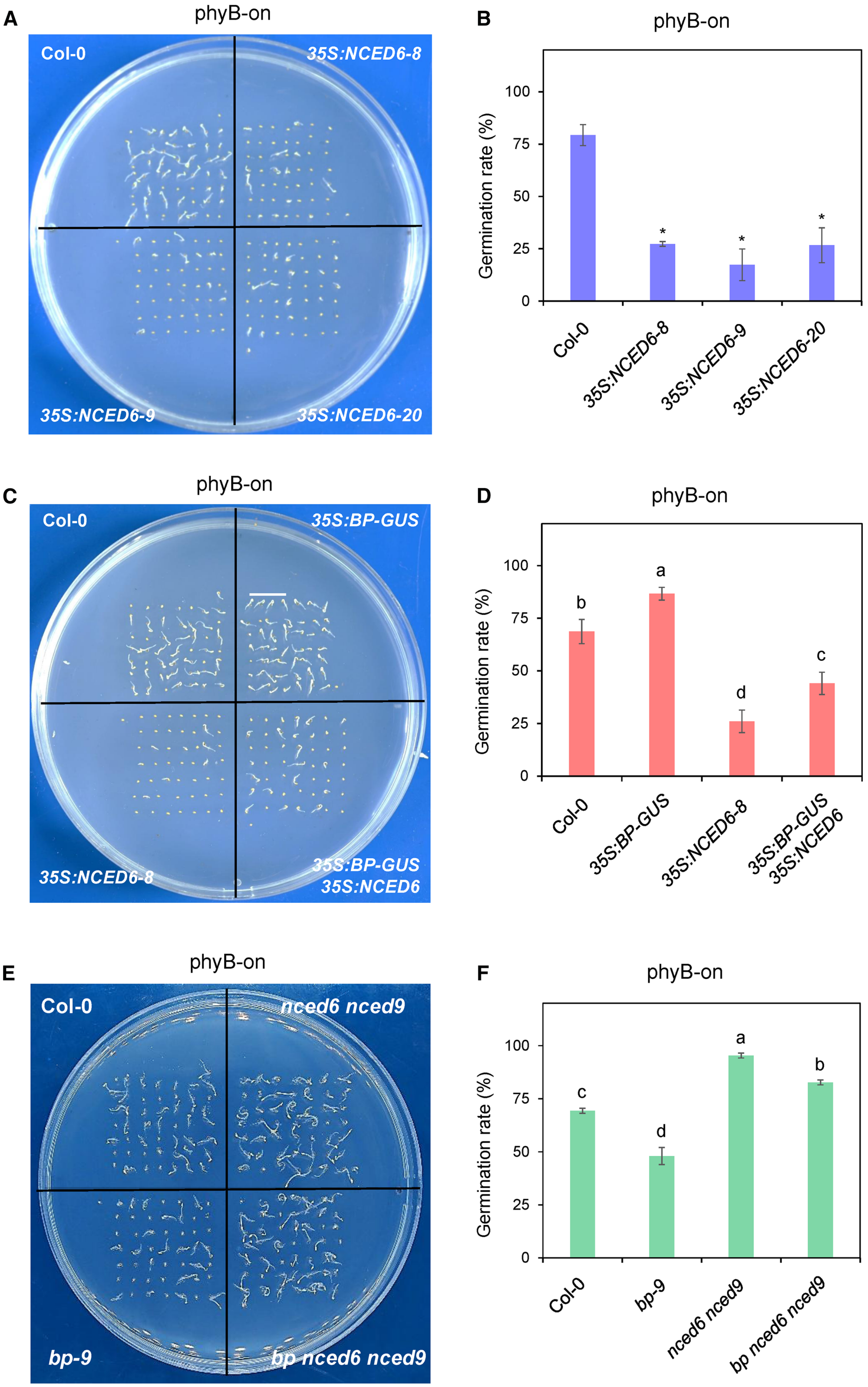

Figure 6. Genetic analysis of *BP* and *NCED6/9* in phyB-dependent seed germination.

(A) Seed germination phenotypes of *35S:NCED6* (*35S:NCED6-GFP*) transgenic lines under phyB-on conditions.

(B) Statistical analysis of the seed germination rates of *35S:NCED6* transgenic lines under phyB-on conditions. Values are shown as mean ± SD (Student's *t*-test, *$p < 0.05$, $n = 3$).

(legend continued on next page)

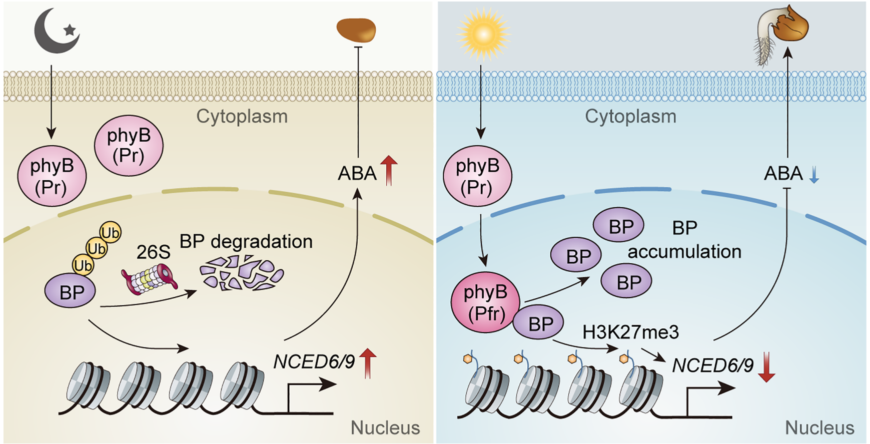

Figure 7. Proposed working model of the phyB-BP-*NCED6/9* cascade in light-initiated seed germination.
In darkness (or FR treatment), BP undergoes ubiquitination and is then degraded via the 26S proteasome. *NCED6* and *NCED9* are highly expressed, leading to increased ABA synthesis and inhibition of germination. Under red light, activated phyB (Pfr form) interacts with and stabilizes BP by reducing its ubiquitination-dependent degradation. Accumulated BP binds to *NCED6/9* and represses their expression by promoting deposition of the repressive histone modification H3K27me3. This regulation decreases ABA levels, thus promoting seed germination.

Col-0, whereas *bp nced6 nced9* seeds germinated at rates closer to *nced6 nced9* than to *bp-9* under phyB-on conditions (Figures 6E and 6F). Taken together, these results indicate that *NCED6* and *NCED9* act epistatically with *BP* in phyB-dependent seed germination.

PIF1 is a master repressor of light-dependent seed germination (Oh et al., 2004); thus, we tested whether BP and PIF1 interact. Yeast cells co-transformed with AD-BP and BD-PIF1 failed to grow on SD/−Leu/−Trp/−His/−Ade dropout medium (Supplemental Figure 12), implying that they do not directly interact. We then examined whether they influence each other's expression in imbibed seeds. RT-qPCR analysis showed that *PIF1* transcript levels were not significantly altered in imbibed *bp-9* seeds compared with Col-0 under either phyB-on or phyB-off conditions (Supplemental Figure 13). In contrast, *BP* transcription was up-regulated in the *PIF1* mutant *pil5-1* at 6 h and 24 h after FR treatment (Supplemental Figure 14), suggesting that PIF1 represses *BP* expression under phyB-off conditions.

DISCUSSION

phyB plays a dominant role in light-initiated seed germination, mainly by modulating GA and ABA metabolism, although the mechanism has remained unclear. Our results establish BP as a positive regulator of light-dependent germination that functions downstream of phyB. BP undergoes ubiquitination and is then degraded by the 26S proteasome; phyB directly interacts with BP and enhances its accumulation via reduced ubiquitination. In turn, BP represses *NCED6* and *NCED9* expression, lowering ABA levels in imbibed seeds in a phyB-dependent manner. Based on these findings, we propose a working model for the phyB-BP-*NCED6/9* module in light-initiated seed germination. Upon exposure to red light, activated phyB interacts with and stabilizes BP via decreased ubiquitination. The accumulated BP binds to and represses *NCED6/9* expression through increased H3K27me3 deposition, which reduces ABA levels and thereby initiates germination (Figure 7).

BP is a new component of light-regulated promotion of seed germination

Light is a major environmental factor governing seed germination. Previous research reported that red light decreases ABA levels by repressing transcription of the ABA biosynthetic genes *NCED6* and *NCED9* and activating the ABA catabolic gene *CYP707A2* (Seo et al., 2006; Oh et al., 2007; Kim et al., 2008). In the present study, phenotypic analyses demonstrated that BP plays a positive role in light-induced germination (Figures 1B, 1C, 1E, and 1G). Genetic evidence suggested that *BP* acts downstream of *phyB* in light-induced seed germination (Figures 1H and 1I). Transcriptional analyses further showed that BP directly binds *NCED6* and *NCED9* both *in vitro* and *in vivo* and represses their expression in imbibed seeds (Figures 4 and 5). These findings suggest that BP promotes light-initiated germination, likely by suppressing *NCED6/9* expression. In addition, higher transcript levels of *NCED6/9* were detected in the *phyB-9* mutant compared with *bp-9* (Supplemental Figure 15), indicating that phyB may repress their expression at least partly through BP.

Beyond their role in regulating *NCED6/9* expression, our transcriptomic analysis revealed that BP and phyB co-regulate the expression of ABA signaling genes (*ABI3*, *ABI4*, and *ABI5*), seed dormancy genes (*DOG1*, *DOGL4*, *RDO5*, *DRM1*, *DRM2*, and *SOM*), and cell wall organization genes (*EXPA1*, *EXPA15*, *EXT21*, *XTH15*, *XTH16*, *XTH4*, *PME18*, *PME25*, and *PME32*). These results suggest that the phyB-BP module serves as an important transcriptional regulator of light-dependent seed germination through diverse pathways.

(C) Seed germination phenotypes of *35S:BP-GUS*, *35S:NCED6-8*, and *35S:BP-GUS 35S:NCED6* lines under phyB-on conditions.
(D) Statistical analysis of the seed germination rates of *35S:BP-GUS*, *35S:NCED6-8*, and *35S:BP-GUS 35S:NCED6* lines under phyB-on conditions.
(E) Seed germination phenotypes of *bp-9*, *nced6 nced9*, and *bp nced6 nced9* lines under phyB-on conditions.
(F) Statistical analysis of the seed germination rates of *bp-9*, *nced6 nced9*, and *bp nced6 nced9* lines under phyB-on conditions.
In **(B)**, **(D)**, and **(F)**, approximately 50 seeds per sample were used for calculations. Values are shown as mean ± SD ($n = 3$). Data were analyzed by one-way ANOVA, followed by Tukey's HSD test. Different letters above bars indicate significant differences ($p < 0.05$).

phyB interacts with BP to transduce light signals in promoting seed germination

The photoreceptor phyB plays a dominant role in promoting seed germination (Shinomura et al., 1994, 1996). It is well established that phytochromes transduce environmental light signals to regulate light-responsive genes via downstream effectors (Leivar and Quail, 2011). A subset of basic helix-loop-helix (bHLH) proteins, the PIF family (PIF1, PIF3, PIF4, PIF5, PIF6, and PIF7), function as negative regulators of phytochrome-mediated responses (Leivar and Quail, 2011). Photoactivated phytochromes translocate from the cytoplasm to the nucleus, where they directly interact with PIF proteins, resulting in rapid phosphorylation and subsequent 26S proteasome-mediated degradation of PIFs (Al-Sady et al., 2008; Bae and Choi, 2008; Lorrain et al., 2008; Shen et al., 2008).

PIF1, a master repressor of light-dependent germination, directly or indirectly regulates the expression of GA- and ABA-related metabolic and signaling genes in imbibed seeds (Oh et al., 2004, 2006, 2007). Additional studies demonstrated that PIF1 activates *NCED6* and *NCED9* through its downstream transcription factor SOM in imbibed seeds (Kim et al., 2008; Park et al., 2011). These findings suggest that the phyB-PIF1-SOM pathway constitutes a critical signal transduction module regulating *NCED6/9* expression. In the present study, our results revealed that phyB also interacts directly with BP (Figure 2). Furthermore, EMSAs and ChIP assays revealed that BP binds directly to *NCED6* and *NCED9* and represses their expression under phyB-on conditions (Figures 4 and 5). These data indicate that the phyB-BP pathway represents an additional transcriptional regulatory module, acting independently of the phyB-PIF1-SOM cascade, to repress *NCED6/9* expression.

Unlike phyB-mediated degradation of PIF1, phyB promoted BP accumulation in imbibed seeds under phyB-on conditions. Immunoblot assays revealed that BP underwent ubiquitination, leading to reduced levels in the *phyB-9* mutant background (Figure 3E). Furthermore, treatment with the 26S proteasome inhibitor MG132 significantly increased BP abundance (Figure 3F). These findings suggest that phyB interacts with BP to decrease its ubiquitination and subsequent proteasome-mediated degradation. RT-qPCR analysis also showed that phyB-on, but not phyB-off, treatment increased BP transcript levels in imbibed seeds (Supplemental Figure 16). Additionally, compared with the wild type, BP transcription was down regulated at 6 and 12 h after phyB-on treatment in the *phyB* mutant (Supplemental Figure 17). Taken together, these results imply that phyB promotes BP accumulation both by decreasing ubiquitination-dependent degradation and by enhancing transcription. Given that *BP* transcripts were elevated in the *pif1* mutant under phyB-off but not phyB-on conditions (Supplemental Figure 14), phyB may promote *BP* expression through PIF1. Further work is needed to elucidate the mechanism underlying phyB-mediated activation of *BP* expression.

A recent study indicated that phyB interacts with the transposase-derived transcription factor Far-red Elongated Hypocotyl 3 (FHY3), leading to the over-accumulation of FHY3 protein in imbibed seeds (Liu et al., 2021). Furthermore, phyA and phyB directly bound to ERF55 and ERF58, displacing them from the promoters of *PIF1* and *SOM* and thus reducing the expression of these genes (Li et al., 2022b). Collectively, these findings suggest that phyB regulates its interacting transcription factors through multiple mechanisms, including alteration of protein stability and modulation of transcriptional activity, to control light-regulated seed germination.

BP represses *NCED6* and *NCED9* by increasing deposition of histone H3K27me3

Reversible histone modifications, including acetylation and methylation, are critical regulators of chromatin structure and gene expression (Liu et al., 2014). Emerging evidence suggests the involvement of epigenetic regulation in light-controlled seed germination. For example, histone deacetylase HDA15 interacts with PIF1 and represses its transcriptional network by reducing histone acetylation at genes related to cell wall loosening and auxin signaling under phyB-off conditions (Gu et al., 2017). HDA15 also directly represses the expression of GA biosynthetic genes *GA20OX1* and *GA20OX2*, thereby suppressing germination under phyB-on conditions (Zheng et al., 2022). Similarly, the histone H3K9 methyltransferase SUVH5 promotes light-mediated germination by repressing ABA pathway-related genes and *DOG1* through increased H3K9me2 deposition in imbibed seeds (Gu et al., 2019). In contrast, the histone H3K27me3 demethylase REF6 directly activates genes related to auxin signaling and cell wall loosening by increasing the levels of H3K27me3 (Wang et al., 2023a). The mutation of EFS, an H3K4 and H3K36 methyltransferase, promoted seed germination even under phyB-off conditions (Lee et al., 2014). Recently, the noncoding RNA *HID1* was reported to repress *NCED9* expression in imbibed seeds by decreasing H3K4me3 levels (Wang et al., 2023b). These studies collectively provide compelling evidence that diverse histone modifications play essential roles in shaping the transcriptional networks that regulate light-dependent germination.

In the present study, relatively higher levels of histone H3K27me3 at *NCED6/9* loci were detected in imbibed *bp-9* seeds compared with wild type seeds, suggesting that BP supresses the expression of these genes by enhancing H3K27me3 deposition. In higher plants, H3K27me3 is catalyzed by Polycomb Repressive Complex 2 (PRC2) (Liu et al., 2010). PRC2 complexes deposit H3K27me3 mainly within genic regions, including proximal promoters and gene bodies, thereby repressing the expression of thousands of genes across the *Arabidopsis* genome (Zhang et al., 2007). Histone modifiers often associate with site-specific transcription factors to regulate the expression of target genes (Liu et al., 2014). BP may thus interact with PRC2 complexes to repress the expression of *NCED* genes under phyB-on conditions. Identification of BP-associated H3K27 methyltransferases and PRC2 components would provide further insight into the transcriptional regulatory role of BP in repressing *NCED6/9*. Additionally, transcription factor dimers have been reported to interact more efficiently with histone-modifying enzymes and co-activator proteins, facilitating the addition or removal of histone modifications (Ortega et al., 2018). In this study, yeast two-hybrid and LCI assays demonstrated that BP can form homodimers (Supplemental Figure 18), which may enhance its interaction with histone modifiers and promote H3K27me3 modification at target genes.

METHODS

Plant materials

Seeds of the *bp-9*, *bp-1*, *phyB-9*, and *pil5-1* mutants, as well as the *35S:BP-GUS* overexpression line, were obtained from the TAIR center (https://www.arabidopsis.org/). The *nced6* mutant (WiscDsLox388C03) (Yang et al., 2022) was obtained from ABRC (https://abrc.osu.edu/), and the *nced9* mutant (SALK_123975C) was purchased from Arashare (https://www.arashare.cn). *BP-GFP-1* and *BP-GFP-2* were generated by introducing the full-length coding sequence (CDS) of *BP*, driven by its native promoter, into a *bp-9* background. *BP-GFP phyB* was constructed by crossing *BP-GFP-1* with *phyB-9*. *35S:phyB* (*35S:phyB-Flag*) transgenic lines were generated using the full-length CDS of *phyB* in a Col-0 background. The *35S:BP-GUS 35S:phyB* line was obtained by crossing *35S:phyB* with *35S:BP-GUS*. *35S:NCED6* (*35S:NCED6-GFP*) transgenic lines were constructed using the full-length CDS of *NCED6* in a Col-0 background. The *35S:BP-GUS 35S:NCED9-GFP* line was produced by crossing *35S:BP-GUS* with *35S:NCED6-GFP-8*. For light-dependent germination, seeds were harvested from plants grown under identical conditions at 22°C with long-day photoperiods (16 h WL/8 h dark). After harvest, seeds were dried in an incubator at 22°C for approximately 1 month prior to germination assays.

phyB-dependent seed germination assays

phyB-dependent seed germination assays were performed as previously described (Oh et al., 2004). Briefly, seeds were surface-sterilized and plated on half-strength Murashige–Skoog (MS; Sigma-Aldrich, USA) medium containing 0.3% sucrose and 1% phytoagar (pH 5.7). Plates were placed in an illuminated incubator under WL (100 μmol m^{-2} s^{-1}) at 22°C. After 1 h of WL exposure, seeds were irradiated with far-red light (3.8 μmol m^{-2} s^{-1}) for 5 min (designated FR or phyB-off) or with far-red light followed by red light (13.1 μmol m^{-2} s^{-1}) for 5 min (designated FR/R or phyB-on). After light treatment, seeds were maintained in darkness for 4 days before germination rates were calculated.

Immunoblot assay

After FR/R treatment, seeds were incubated in darkness for the indicated times. Total proteins were extracted using lysis buffer (50 mM Tris-HCl [pH 7.4], 150 mM NaCl, 2 mM $MgCl_2$, 1 mM dithiothreitol, 20% glycerol, 1% NP-40, 2 mM phenylmethylsulfonyl fluoride, and a protease inhibitor cocktail [Roche, Basel, Switzerland]). BP protein in *35S:BP-GUS* and *35S:BP-GUS phyB* seeds was immunoprecipitated with an anti-GUS antibody (Abmart, Shanghai, China). BP protein levels and ubiquitination status were then detected using an anti-ubiquitin antibody (PTM Biolabs, Hangzhou, China).

coIP assay

Seeds of *35S:BP-GUS* and *35S:BP-GUS phyB-9* were treated with either FR or FR/R, then incubated in darkness for 24 h. Total protein was extracted with lysis buffer and centrifuged twice at 15 000 *g* for 15 min at 4°C. Extracts were incubated with an anti-GUS antibody for 3 h at 4°C, then mixed with 25 μl protein A agarose beads (Santa Cruz, CA, USA) for 1 h at 4°C. Beads were washed three times with wash buffer (50 mM Tris-HCl [pH 7.4], 150 mM NaCl, 2 mM $MgCl_2$, 1 mM dithiothreitol, 10% glycerol, 1% NP-40, 2 mM PMSF, and a protease inhibitor cocktail [Roche]). Precipitated proteins were separated via 10% SDS-PAGE and detected with an anti-phyB antibody (phytoAB, San Francisco, CA, USA).

LCI assay

The firefly LCI assay was performed in leaves of 42-day-old tobacco (*Nicotiana benthamiana*) plants. The full-length CDS of *phyB* and *BP* fused to cLUC or nLUC were transformed into *Agrobacterium tumefaciens* (strain GV3101). The bacterial suspensions were co-infiltrated into tobacco leaves using a needleless syringe. After 2 days of incubation at 22°C, the luciferase substrate (Promega, Madison, WI, USA) was infiltrated into the leaves; luminescence images were immediately captured using a low-light cooled charge-coupled device imaging system (Bio-Rad Laboratories, Hercules, CA, USA). Each experiment was repeated at least three times. Data were quantified using ImageJ software (National Institutes of Health, Bethesda, MD, USA).

Yeast two-hybrid assay

For yeast two-hybrid assays, the full-length CDS of *phyB* and *BP* fused with pGADT7-AD or pGBKT7-BD were co-transformed into *Saccharomyces cerevisiae* strain AH109 (BD Clontech, Palo Alto, CA, USA). Transformants were first plated on SD/−Trp/−Leu dropout medium, then transferred to SD/−Trp/−Leu/−His/−Ade dropout medium for interaction analysis.

RNA extraction and qRT-PCR assay

After FR/R treatment, seeds (~0.1 g) were incubated in darkness at 22°C for 0, 12, and 24 h. Total RNA was extracted using TRIzol reagent (Invitrogen, Carlsbad, CA, USA), in accordance with the manufacturer's protocol. After DNase I treatment, first-strand cDNA was synthesized from 2 μg of total RNA using the TransScript One-Step gDNA Removal and cDNA Synthesis SuperMix Kit (TransGen, Beijing, China) according to the manufacturer's instructions. Quantitative PCR reactions were performed in a total volume of 20 μl containing 0.5 μl of each primer (100 nM) and 10 μl of SYBR Green PCR Supermix (Bio-Rad Laboratories) on an ABI7500 Real-Time PCR System (Applied Biosystems, Foster City, CA, USA). The thermal cycling program consisted of initial denaturation at 94°C for 3 min, followed by 40 cycles of 94°C for 5 s and 60°C for 1 min. All reactions were normalized to Ct values of the reference gene *PP2A*. Relative expression levels were calculated using the $2^{-\Delta\Delta Ct}$ method. Values represent the mean of three biological replicates. Gene-specific primer sequences are provided in Supplemental Table 2.

RNA-seq assay

Total RNA was extracted as described above, and mRNA-seq libraries were constructed using an mRNA Seq Kit (Illumina, San Diego, CA, USA). RNA-seq was performed by Millennium Genomics (Shenzhen, China) with three biological replicates. High-quality clean reads were obtained by removing adaptor sequences, ambiguous reads (N > 10%), and low-quality reads in which more than 50% of bases had a quality value (*Q*) ≤ 5. Clean reads were mapped to the *Arabidopsis* genome (TAIR10) using HISAT2 with default settings (Pertea et al., 2016). Differentially expressed genes were identified with Cuffdiff (Trapnell et al., 2010), and those with fold-change values > 1.5 ($p < 0.05$) were selected.

EMSA

To generate the BP-His construct, the full-length CDS of *BP* was cloned into the pET32a vector. His and BP-His proteins were expressed in *E. coli* BL21 (DE3) and induced by isopropyl β-D-thiogalactopyranoside (IPTG). Proteins were purified using HisSep Ni-NTA Agarose Resin (Yeasen, Shanghai, China), in accordance with the manufacturer's instructions. EMSA experiments were carried out using the LightShift Chemiluminescent EMSA kit (Thermo Scientific, Waltham, MA, USA). Briefly, 0.5 μg of BP-His was incubated with biotin-labeled probes from *NCED6* and *NCED9* for 20 min at room temperature. Protein–DNA complexes were separated on 6% polyacrylamide gels. Oligonucleotide probes corresponding to the exon of *NCED6* (430–473 bp) and promoter of *NCED9* (−1261 to −1211 bp) were synthesized and biotin-labeled (Sango Biotech, Shanghai, China). All vector construction primers and oligonucleotide sequences are listed in Supplemental Table 2.

Measurement of endogenous ABA content

ABA content was measured as previously reported (Gu et al., 2019). Finely powdered tissue (~30 mg fresh weight) was extracted with ethyl acetate by vortexing, followed by ultrasonication in ice-cold water. Before ultrasonication, 1 ng of [$^2H_6$]ABA was added as an internal standard. After centrifugation, supernatants were collected and evaporated under nitrogen. Residues were dissolved in methanol and filtered through a 0.22-μm

membrane. Samples were analyzed by ultra-performance liquid chromatography coupled with quadrupole time-of-flight mass spectrometry (UPLC–QTOF–MS; Acquity UPLC I-Class/Xevo G2-XS QTOF, Waters Corporation, Milford, MA, USA). Quantitative analysis was performed using a calibration curve generated by plotting ABA standard concentrations against the peak area of [$^2H_6$]ABA. Each sample was assayed in triplicate.

Dual-luciferase reporter assay

The dual-luciferase reporter assay was performed as previously described (Ma et al., 2020, 2023). The *NCED6* exon and *NCED9* promoter were cloned into the pGreenII 0800-LUC vector; the CDS of *BP* was inserted into the pGreenII 62-SK vector. Each effector–reporter pair was transiently co-expressed in tobacco leaves using an *Agrobacterium*-mediated system, as in the LCI assay. LUC and REN luciferase activities were measured with the Dual-Luciferase Reporter Assay System (Promega). Each assay was performed in six independent replicates.

ChIP assay

ChIP assays were performed as previously described (Gu et al., 2017), with minor modifications. Seeds ($\sim$0.2–0.3 g) were cross-linked with 1% formaldehyde under vacuum for 1 h. Chromatin was isolated, fragmented to an average size of 500 bp by sonication, and immunoprecipitated with anti-GFP (Abcam, Cambridge, UK), anti-H3K4me3 (Millipore, Boston, MA, USA), or anti-H3K27me3 (Millipore) antibodies. Cross-links were then reversed, and the quantity of immunoprecipitated DNA fragments was measured by quantitative PCR with gene-specific primers (Supplemental Table 2).

Statistical analysis

Student's *t*-test was used for statistical comparison of differences between two groups. For comparisons among more than two groups, one-way analysis of variance (ANOVA) followed by Tukey's honestly significant difference (HSD) test was applied. Values of $p < 0.05$ were considered statistically significant. Data are presented as mean ± standard deviation (SD).

DATA AND CODE AVAILABILITY

The raw RNA-seq data of Col-0 and *bp-9* have been deposited in the National Center for Biotechnology Information (NCBI) database (https://www.ncbi.nlm.nih.gov/bioproject) under BioProject accession number PRJNA489162.

FUNDING

This work was supported by grants from the National Natural Science Foundation of China (Nos. 32571474 and 31801091), the Tianshan Talent in Science and Technology Innovation Team (No. 2024TSYCTD0010), and the Youth Innovation Promotion Association, Chinese Academy of Sciences (No. 201860).

ACKNOWLEDGMENTS

We thank Dr. Xiaochao Chen from Xianghu Lab for his kind assistance in providing *nced6* mutant seeds. We are also grateful to the anonymous reviewers for their constructive comments on this study. The authors declare no conflicts of interest.

AUTHOR CONTRIBUTIONS

X.L. conceived the research; D.G. performed the RNA-seq assays, examined the phenotype, and generated the transgenic lines; Y.W. detected the interaction and performed the ChIP-qPCR and immunoblot assays. M.Z. analyzed the germination phenotype, quantified gene expression levels, and conducted the dual-luciferase reporter assays. H.C. and L.D. conducted the EMSA assays. S.W. measured the ABA levels. R.J. analyzed the expression patterns. X.J. performed the cell-free degradation assays. J.X. and F.Z. screened the mutants and transgenic lines and cultivated the plant materials. X.L. analyzed the data and wrote the article.

SUPPLEMENTAL INFORMATION

Supplemental information is available at *Plant Communications Online*.

Received: January 13, 2025
Revised: August 7, 2025
Accepted: September 3, 2025
Published: September 4, 2025

REFERENCES

Al-Sady, B., Kikis, E.A., Monte, E., and Quail, P.H. (2008). Mechanistic duality of transcription factor function in phytochrome signaling. Proc. Natl. Acad. Sci. USA **105**:2232–2237. https://doi.org/10.1073/pnas.0711675105.

Al-Sady, B., Ni, W., Kircher, S., Schäfer, E., and Quail, P.H. (2006). Photoactivated phytochrome induces rapid PIF3 phosphorylation prior to proteasome-mediated degradation. Mol. Cell **23**:439–446. https://doi.org/10.1016/j.molcel.2006.06.011.

Bae, G., and Choi, G. (2008). Decoding of light signals by plant phytochromes and their interacting proteins. Annu. Rev. Plant Biol. **59**:281–311. https://doi.org/10.1146/annurev.arplant.59.032607.092859.

Bolduc, N., and Hake, S. (2009). The maize transcription factor KNOTTED1 directly regulates the gibberellin catabolism gene ga2ox1. Plant Cell **21**:1647–1658. https://doi.org/10.1105/tpc.109.068221.

Byrne, M.E., Simorowski, J., and Martienssen, R.A. (2002). ASYMMETRIC LEAVES1 reveals knox gene redundancy in Arabidopsis. Development **129**:1957–1965. https://doi.org/10.1242/dev.129.8.1957.

Carrera-Castano, G., Calleja-Cabrera, J., Pernas, M., Gomez, L., and Onate-Sanchez, L. (2020). An Updated Overview on the Regulation of Seed Germination. Plants **9**:703. https://doi.org/10.3390/plants9060703.

Douglas, S.J., Chuck, G., Dengler, R.E., Pelecanda, L., and Riggs, C.D. (2002). KNAT1 and ERECTA regulate inflorescence architecture in Arabidopsis. Plant Cell **14**:547–558. https://doi.org/10.1105/tpc.010391.

Finch-Savage, W.E., and Leubner-Metzger, G. (2006). Seed dormancy and the control of germination. New Phytol. **171**:501–523. https://doi.org/10.1111/j.1469-8137.2006.01787.x.

Finkelstein, R.R., and Lynch, T.J. (2000). The Arabidopsis abscisic acid response gene ABI5 encodes a basic leucine zipper transcription factor. Plant Cell **12**:599–609. https://doi.org/10.1105/tpc.12.4.599.

Finkelstein, R.R., Wang, M.L., Lynch, T.J., Rao, S., and Goodman, H.M. (1998). The Arabidopsis abscisic acid response locus ABI4 encodes an APETALA 2 domain protein. Plant Cell **10**:1043–1054. https://doi.org/10.1105/tpc.10.6.1043.

Frey, A., Effroy, D., Lefebvre, V., Seo, M., Perreau, F., Berger, A., Sechet, J., To, A., North, H.M., and Marion-Poll, A. (2012). Epoxycarotenoid cleavage by NCED5 fine-tunes ABA accumulation and affects seed dormancy and drought tolerance with other NCED family members. Plant J. **70**:501–512. https://doi.org/10.1111/j.1365-313X.2011.04887.x.

Giraudat, J., Hauge, B.M., Valon, C., Smalle, J., Parcy, F., and Goodman, H.M. (1992). Isolation of the Arabidopsis ABI3 gene by positional cloning. Plant Cell **4**:1251–1261. https://doi.org/10.1105/tpc.4.10.1251.

Gu, D., Chen, C.Y., Zhao, M., Zhao, L., Duan, X., Duan, J., Wu, K., and Liu, X. (2017). Identification of HDA15-PIF1 as a key repression module directing the transcriptional network of seed germination in the dark. Nucleic Acids Res. **45**:7137–7150. https://doi.org/10.1093/nar/gkx283.

Gu, D., Ji, R., He, C., Peng, T., Zhang, M., Duan, J., Xiong, C., and Liu, X. (2019). Arabidopsis Histone Methyltransferase SUVH5 Is a Positive Regulator of Light-Mediated Seed Germination. Front. Plant Sci. **10**:841. https://doi.org/10.3389/fpls.2019.00841.

Kami, C., Lorrain, S., Hornitschek, P., and Fankhauser, C. (2010). Light-regulated plant growth and development. Curr. Top. Dev. Biol. **91**:29–66. https://doi.org/10.1016/S0070-2153(10)91002-8.

Kim, D.H., Yamaguchi, S., Lim, S., Oh, E., Park, J., Hanada, A., Kamiya, Y., and Choi, G. (2008). SOMNUS, a CCCH-type zinc finger protein in Arabidopsis, negatively regulates light-dependent seed germination downstream of PIL5. Plant Cell **20**:1260–1277. https://doi.org/10.1105/tpc.108.058859.

Lee, N., Kang, H., Lee, D., and Choi, G. (2014). A histone methyltransferase inhibits seed germination by increasing PIF1 mRNA expression in imbibed seeds. Plant J. **78**:282–293. https://doi.org/10.1111/tpj.12467.

Lefebvre, V., North, H., Frey, A., Sotta, B., Seo, M., Okamoto, M., Nambara, E., and Marion-Poll, A. (2006). Functional analysis of Arabidopsis NCED6 and NCED9 genes indicates that ABA synthesized in the endosperm is involved in the induction of seed dormancy. Plant J. **45**:309–319. https://doi.org/10.1111/j.1365-313X.2005.02622.x.

Leivar, P., and Quail, P.H. (2011). PIFs: pivotal components in a cellular signaling hub. Trends Plant Sci. **16**:19–28. https://doi.org/10.1016/j.tplants.2010.08.003.

Li, J., Li, G., Wang, H., and Wang Deng, X. (2011). Phytochrome signaling mechanisms. Arabidopsis Book **9**:e0148. https://doi.org/10.1199/tab.0148.

Li, Z., Luo, X., Wang, L., and Shu, K. (2022a). ABSCISIC ACID INSENSITIVE 5 mediates light-ABA/gibberellin crosstalk networks during seed germination. J. Exp. Bot. **73**:4674–4682. https://doi.org/10.1093/jxb/erac200.

Li, Z., Sheerin, D.J., von Roepenack-Lahaye, E., Stahl, M., and Hiltbrunner, A. (2022b). The phytochrome interacting proteins ERF55 and ERF58 repress light-induced seed germination in Arabidopsis thaliana. Nat. Commun. **13**:1656. https://doi.org/10.1038/s41467-022-29315-3.

Lincoln, C., Long, J., Yamaguchi, J., Serikawa, K., and Hake, S. (1994). A knotted1-like homeobox gene in Arabidopsis is expressed in the vegetative meristem and dramatically alters leaf morphology when overexpressed in transgenic plants. Plant Cell **6**:1859–1876. https://doi.org/10.1105/tpc.6.12.1859.

Liu, C., Lu, F., Cui, X., and Cao, X. (2010). Histone methylation in higher plants. Annu. Rev. Plant Biol. **61**:395–420. https://doi.org/10.1146/annurev.arplant.043008.091939.

Liu, S., Yang, L., Li, J., Tang, W., Li, J., and Lin, R. (2021). FHY3 interacts with phytochrome B and regulates seed dormancy and germination. Plant Physiol. **187**:289–302. https://doi.org/10.1093/plphys/kiab147.

Liu, X., Yang, S., Zhao, M., Luo, M., Yu, C.W., Chen, C.Y., Tai, R., and Wu, K. (2014). Transcriptional repression by histone deacetylases in plants. Mol. Plant **7**:764–772. https://doi.org/10.1093/mp/ssu033.

Lorrain, S., Allen, T., Duek, P.D., Whitelam, G.C., and Fankhauser, C. (2008). Phytochrome-mediated inhibition of shade avoidance involves degradation of growth-promoting bHLH transcription factors. Plant J. **53**:312–323. https://doi.org/10.1111/j.1365-313X.2007.03341.x.

Ma, X., Yuan, Y., Wu, Q., Wang, J., Li, J., and Zhao, M. (2020). LcEIL2/3 are involved in fruitlet abscission via activating genes related to ethylene biosynthesis and cell wall remodeling in litchi. Plant J. **103**:1338–1350. https://doi.org/10.1111/tpj.14804.

Ma, X., Xie, X., He, Z., Wang, F., Fan, R., Chen, Q., Zhang, H., Huang, Z., Wu, H., Zhao, M., and Li, J. (2023). A LcDOF5.6-LcRbohD regulatory module controls the reactive oxygen species-mediated fruitlet abscission in litchi. Plant J. **113**:954–968. https://doi.org/10.1111/tpj.16092.

Martinez-Andujar, C., Ordiz, M.I., Huang, Z., Nonogaki, M., Beachy, R. N., and Nonogaki, H. (2011). Induction of 9-cis-epoxycarotenoid dioxygenase in Arabidopsis thaliana seeds enhances seed dormancy. Proc. Natl. Acad. Sci. USA **108**:17225–17229. https://doi.org/10.1073/pnas.1112151108.

Oh, E., Kim, J., Park, E., Kim, J.I., Kang, C., and Choi, G. (2004). PIL5, a phytochrome-interacting basic helix-loop-helix protein, is a key negative regulator of seed germination in Arabidopsis thaliana. Plant Cell **16**:3045–3058. https://doi.org/10.1105/tpc.104.025163.

Oh, E., Yamaguchi, S., Kamiya, Y., Bae, G., Chung, W.I., and Choi, G. (2006). Light activates the degradation of PIL5 protein to promote seed germination through gibberellin in Arabidopsis. Plant J. **47**:124–139. https://doi.org/10.1111/j.1365-313X.2006.02773.x.

Oh, E., Yamaguchi, S., Hu, J., Yusuke, J., Jung, B., Paik, I., Lee, H.S., Sun, T.P., Kamiya, Y., and Choi, G. (2007). PIL5, a phytochrome-interacting bHLH protein, regulates gibberellin responsiveness by binding directly to the GAI and RGA promoters in Arabidopsis seeds. Plant Cell **19**:1192–1208. https://doi.org/10.1105/tpc.107.050153.

Ori, N., Eshed, Y., Chuck, G., Bowman, J.L., and Hake, S. (2000). Mechanisms that control knox gene expression in the Arabidopsis shoot. Development **127**:5523–5532. https://doi.org/10.1242/dev.127.24.5523.

Ortega, E., Rengachari, S., Ibrahim, Z., Hoghoughi, N., Gaucher, J., Holehouse, A.S., Khochbin, S., and Panne, D. (2018). Transcription factor dimerization activates the p300 acetyltransferase. Nature **562**:538–544. https://doi.org/10.1038/s41586-018-0621-1.

Park, J., Lee, N., Kim, W., Lim, S., and Choi, G. (2011). ABI3 and PIL5 collaboratively activate the expression of SOMNUS by directly binding to its promoter in imbibed Arabidopsis seeds. Plant Cell **23**:1404–1415. https://doi.org/10.1105/tpc.110.080721.

Pertea, M., Kim, D., Pertea, G.M., Leek, J.T., and Salzberg, S.L. (2016). Transcript-level expression analysis of RNA-seq experiments with HISAT, StringTie and Ballgown. Nat. Protoc. **11**:1650–1667. https://doi.org/10.1038/nprot.2016.095.

Ragni, L., Belles-Boix, E., Günl, M., and Pautot, V. (2008). Interaction of KNAT6 and KNAT2 with BREVIPEDICELLUS and PENNYWISE in Arabidopsis inflorescences. Plant Cell **20**:888–900. https://doi.org/10.1105/tpc.108.058230.

Reed, J.W., Nagpal, P., Poole, D.S., Furuya, M., and Chory, J. (1993). Mutations in the gene for the red/far-red light receptor phytochrome B alter cell elongation and physiological responses throughout Arabidopsis development. Plant Cell **5**:147–157. https://doi.org/10.1105/tpc.5.2.147.

Seo, M., and Koshiba, T. (2002). Complex regulation of ABA biosynthesis in plants. Trends Plant Sci. **7**:41–48. https://doi.org/10.1016/s1360-1385(01)02187-2.

Seo, M., Nambara, E., Choi, G., and Yamaguchi, S. (2009). Interaction of light and hormone signals in germinating seeds. Plant Mol. Biol. **69**:463–472. https://doi.org/10.1007/s11103-008-9429-y.

Seo, M., Hanada, A., Kuwahara, A., Endo, A., Okamoto, M., Yamauchi, Y., North, H., Marion-Poll, A., Sun, T.P., Koshiba, T., et al. (2006). Regulation of hormone metabolism in Arabidopsis seeds: phytochrome regulation of abscisic acid metabolism and abscisic acid regulation of gibberellin metabolism. Plant J. **48**:354–366. https://doi.org/10.1111/j.1365-313X.2006.02881.x.

Shen, H., Zhu, L., Castillon, A., Majee, M., Downie, B., and Huq, E. (2008). Light-induced phosphorylation and degradation of the negative regulator PHYTOCHROME-INTERACTING FACTOR1 from Arabidopsis depend upon its direct physical interactions with photoactivated phytochromes. Plant Cell **20**:1586–1602. https://doi.org/10.1105/tpc.108.060020.

Shen, Y., Khanna, R., Carle, C.M., and Quail, P.H. (2007). Phytochrome induces rapid PIF5 phosphorylation and degradation in response to

red-light activation. Plant Physiol. **145**:1043–1051. https://doi.org/10.1104/pp.107.105601.

Shinomura, T., Nagatani, A., Chory, J., and Furuya, M. (1994). The Induction of Seed Germination in Arabidopsis thaliana Is Regulated Principally by Phytochrome B and Secondarily by Phytochrome A. Plant Physiol. **104**:363–371. https://doi.org/10.1104/pp.104.2.363.

Shinomura, T., Nagatani, A., Hanzawa, H., Kubota, M., Watanabe, M., and Furuya, M. (1996). Action spectra for phytochrome A- and B-specific photoinduction of seed germination in Arabidopsis thaliana. Proc. Natl. Acad. Sci. USA **93**:8129–8133. https://doi.org/10.1073/pnas.93.15.8129.

Smith, H.M.S., Boschke, I., and Hake, S. (2002). Selective interaction of plant homeodomain proteins mediates high DNA-binding affinity. Proc. Natl. Acad. Sci. USA **99**:9579–9584. https://doi.org/10.1073/pnas.092271599.

Toyomasu, T., Kawaide, H., Mitsuhashi, W., Inoue, Y., and Kamiya, Y. (1998). Phytochrome regulates gibberellin biosynthesis during germination of photoblastic lettuce seeds. Plant Physiol. **118**:1517–1523. https://doi.org/10.1104/pp.118.4.1517.

Trapnell, C., Williams, B.A., Pertea, G., Mortazavi, A., Kwan, G., van Baren, M.J., Salzberg, S.L., Wold, B.J., and Pachter, L. (2010). Transcript assembly and quantification by RNA-Seq reveals unannotated transcripts and isoform switching during cell differentiation. Nat. Biotechnol. **28**:511–515. https://doi.org/10.1038/nbt.1621.

Wang, Y., Gu, D., Deng, L., He, C., Zheng, F., and Liu, X. (2023a). The Histone H3K27 Demethylase REF6 Is a Positive Regulator of Light-Initiated Seed Germination in Arabidopsis. Cells **12**:295. https://doi.org/10.3390/cells12020295.

Wang, Y., Fan, Y., Fan, D., Zhou, X., Jiao, Y., Deng, X.W., and Zhu, D. (2023b). The noncoding RNA HIDDEN TREASURE 1 promotes phytochrome B-dependent seed germination by repressing abscisic acid biosynthesis. Plant Cell **35**:700–716. https://doi.org/10.1093/plcell/koac334.

Woerlen, N., Allam, G., Popescu, A., Corrigan, L., Pautot, V., and Hepworth, S.R. (2017). Repression of BLADE-ON-PETIOLE genes by KNOX homeodomain protein BREVIPEDICELLUS is essential for differentiation of secondary xylem in Arabidopsis root. Planta **245**:1079–1090. https://doi.org/10.1007/s00425-017-2663-2.

Yang, D., Zhao, F., Zhu, D., Chen, X., Kong, X., Wu, Y., Chen, M., Du, J., Qu, L.J., and Wu, Z. (2022). Progressive chromatin silencing of ABA biosynthesis genes permits seed germination in Arabidopsis. Plant Cell **34**:2871–2891. https://doi.org/10.1093/plcell/koac134.

Yang, L., Liu, S., and Lin, R. (2020). The role of light in regulating seed dormancy and germination. J. Integr. Plant Biol. **62**:1310–1326. https://doi.org/10.1111/jipb.13001.

Zhang, X., Clarenz, O., Cokus, S., Bernatavichute, Y.V., Pellegrini, M., Goodrich, J., and Jacobsen, S.E. (2007). Whole-genome analysis of histone H3 lysine 27 trimethylation in Arabidopsis. PLoS Biol. **5**:e129. https://doi.org/10.1371/journal.pbio.0050129.

Zhao, M., Yang, S., Chen, C.Y., Li, C., Shan, W., Lu, W., Cui, Y., Liu, X., and Wu, K. (2015). Arabidopsis BREVIPEDICELLUS interacts with the SWI2/SNF2 chromatin remodeling ATPase BRAHMA to regulate KNAT2 and KNAT6 expression in control of inflorescence architecture. PLoS Genet. **11**:e1005125. https://doi.org/10.1371/journal.pgen.1005125.

Zheng, F., Wang, Y., Gu, D., and Liu, X. (2022). Histone Deacetylase HDA15 Restrains PHYB-Dependent Seed Germination via Directly Repressing GA20ox1/2 Gene Expression. Cells **11**:3788. https://doi.org/10.3390/cells11233788.

Zhou, Y., Zhou, B., Pache, L., Chang, M., Khodabakhshi, A.H., Tanaseichuk, O., Benner, C., and Chanda, S.K. (2019). Metascape provides a biologist-oriented resource for the analysis of systems-level datasets. Nat. Commun. **10**:1523. https://doi.org/10.1038/s41467-019-09234-6.

Plant Communications, Volume *6*

Supplemental information

Phytochrome B stabilizes the KNOX transcription factor BP/KNAT1 to promote light-initiated seed germination in *Arabidopsis thaliana*

Dachuan Gu, Yahan Wang, Minglei Zhao, Hangcong Chen, Shuhua Wu, Xia Jin, Ling Deng, Rujun Ji, Jingyan Xu, Feng Zheng, and Xuncheng Liu

Supplemental Information

Phytochrome B stabilizes the KNOX transcription factor BP/KNAT1 to promote light-initiated seed germination in *Arabidopsis thaliana*

Dachuan Gu[1,2,5], Yahan Wang[1,2,5], Minglei Zhao[3,5], Hangcong Chen[1,2], Suhua Wu[4], Xia Jin[1,2], Ling Deng[1,2], Rujun Ji[1,2], Jinyan Xu[1,2], Feng Zheng[1], Xuncheng Liu[1,2,*]

[1]Guangdong Provincial Key Laboratory of Applied Botany, Key Laboratory of South China Agricultural Plant Molecular Analysis and Genetic Improvement, South China Botanical Garden, Chinese Academy of Sciences, Guangzhou, 510650, China
[2]College of Life Sciences, University of Chinese Academy of Sciences, Beijing, 100049, China
[3]College of Horticulture, South China Agricultural University, Guangzhou, China
[4]Guangdong Provincial Key Laboratory of Tea Plant Resources Innovation and Utilization, Tea Research Institute, Guangdong Academy of Agricultural Sciences, Guangzhou 510640, China
[5]These authors contributed equally to this work

[*]Corresponding author: Xuncheng Liu (xunchengliu@scbg.ac.cn)

Running title: phyB stabilizes BP/KNAT1 to promote seed germination

Short summary: KNOX-type transcription factor BP/ KNAT1 plays a positive role in light-dependent seed germination. BP directly represses the expression of ABA biosynthetic genes *NCED6/9*. The red/far-red light photoreceptor phyB interacts with and stabilizes BP in imbibed seeds. This work reveals that phyB-BP-NCED6/9 cascade promotes light-initiated seed germination by repressing ABA biosynthesis.

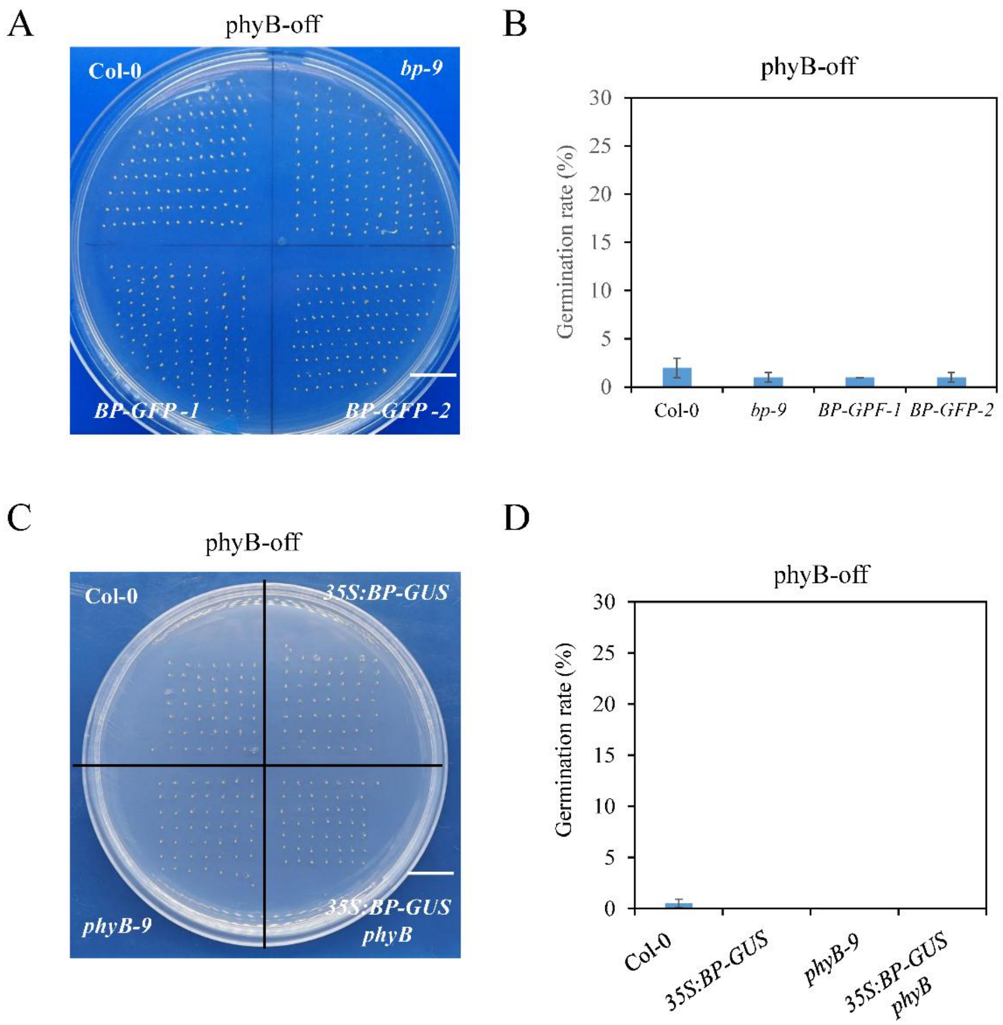

Supplemental Figure 1. Germination phenotype of the *bp-9* mutant, *BP-GFP* complementary lines, *35S:BP-GUS*, and *35S:BP-GUS phyB-9* seeds in the phyB-off condition. (A, B) Germination patterns of Col-0, the *bp-9* mutant, and *BP* transgenic complement lines (*BP-GFP-1* and *-2*) in the phyB-off condition. (C, D) Germination patterns and rates of Col-0, *35S:BP*, *phyB-9*, and *35S:BP phyB* seeds in the phyB-off condition. About 50 seeds of each sample were used for calculations. The experiment was performed in triplicate. Scale bar: 1 cm.

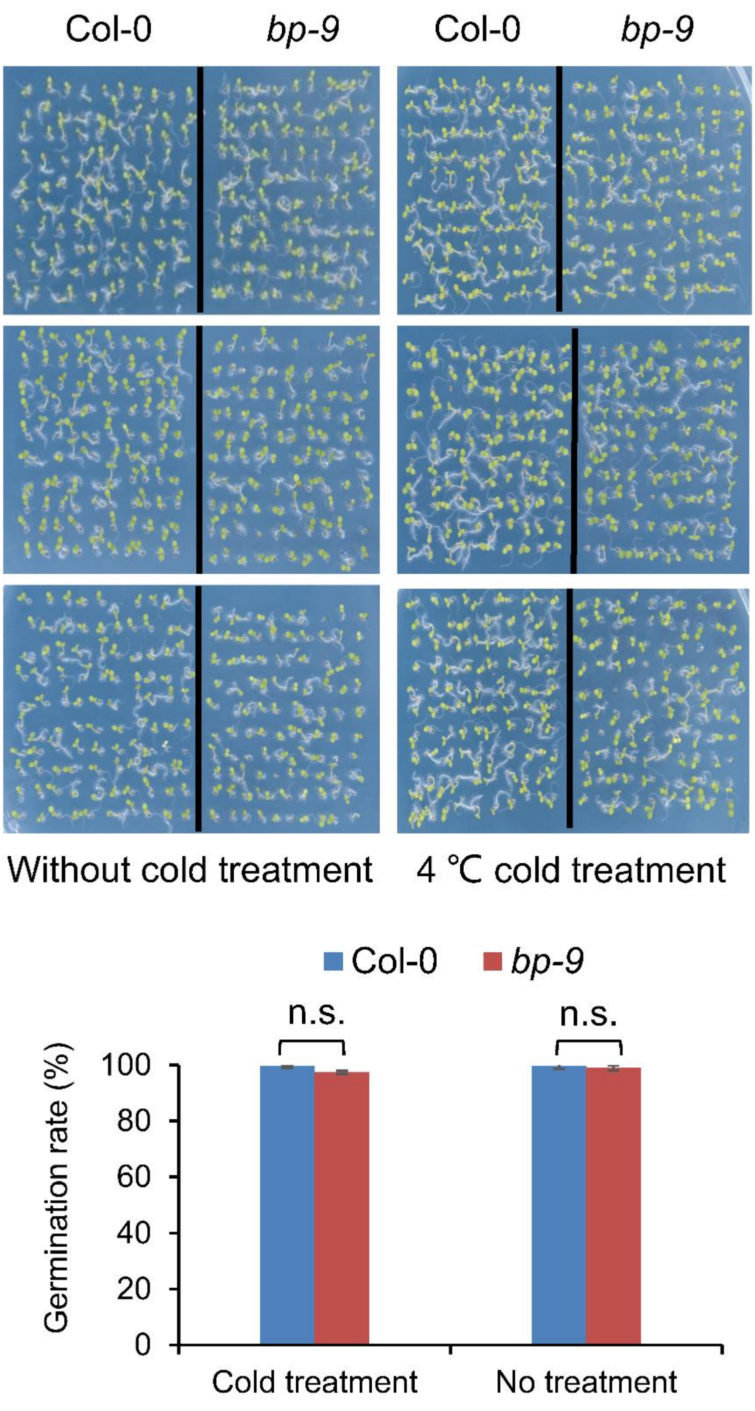

Supplemental Figure 2. Analysis of the effect of stratification treatment (or not) on the germination of *bp-9* mutant. The freshly harvested seeds of Col-0 and *bp-9* were surface sterilized and plated on 0.6% (w/v) agar (pH 5.7) within 1 h. Seeds were either stratified or not at 4 °C for 4 d, followed by incubation in white light (80 μmol $m^{-2}s^{-1}$) for 4 d and scoring of the germination rates. Values are shown as means ± SD (Student's *t*-test, * $p < 0.05$, n=3), n.s. indicate no significant difference.

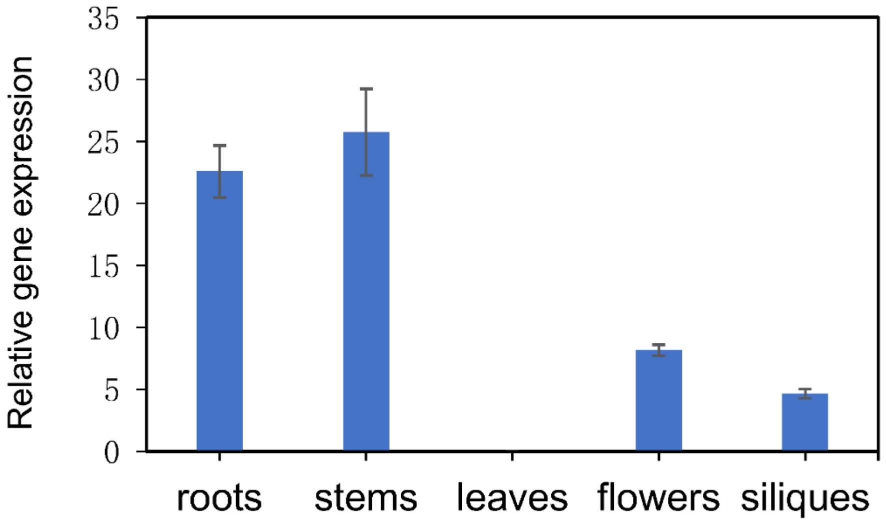

Supplemental Figure 3. RT-qPCR analysis of the transcription levels of *BP* in multiple tissues including roots, stems, leaves, flowers, and siliques of 35-day-old plants. *PP2A* was used as internal control. Values are shown as means ± SD (n=3).

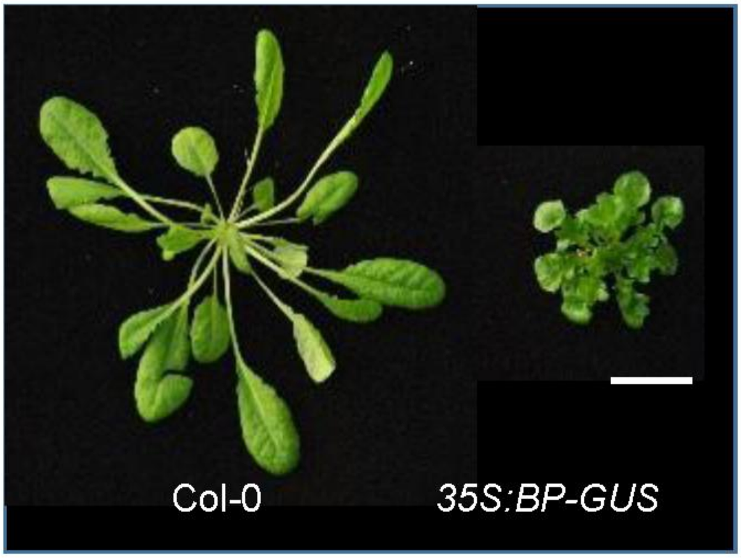

Supplemental Figure 4. Phenotypes of 20-day-od *35S:BP-GUS* transgenic line. Scale bar, 2cm.

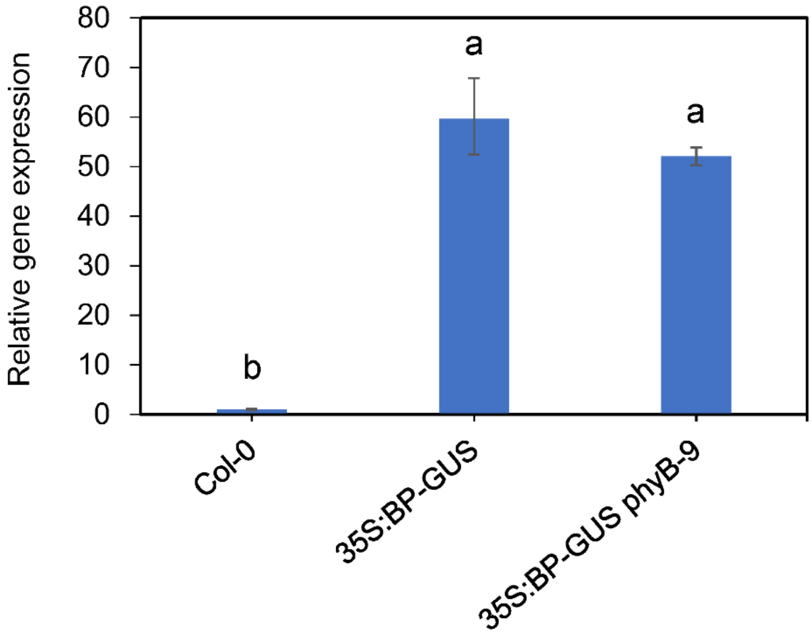

Supplemental Figure 5. RT-qPCR analysis of the expression levels of *BP* in imbibed Col-0, *35S:BP-GUS* and *35S:BP-GUS phyB-9* seeds under phyB-on conditions. *PP2A* was used as internal control. Values are shown as means ± SD. The data were analyzed by one-way ANOVA followed by Tukey's HSD test. Different letters above bars indicate significant differences ($p < 0.05$).

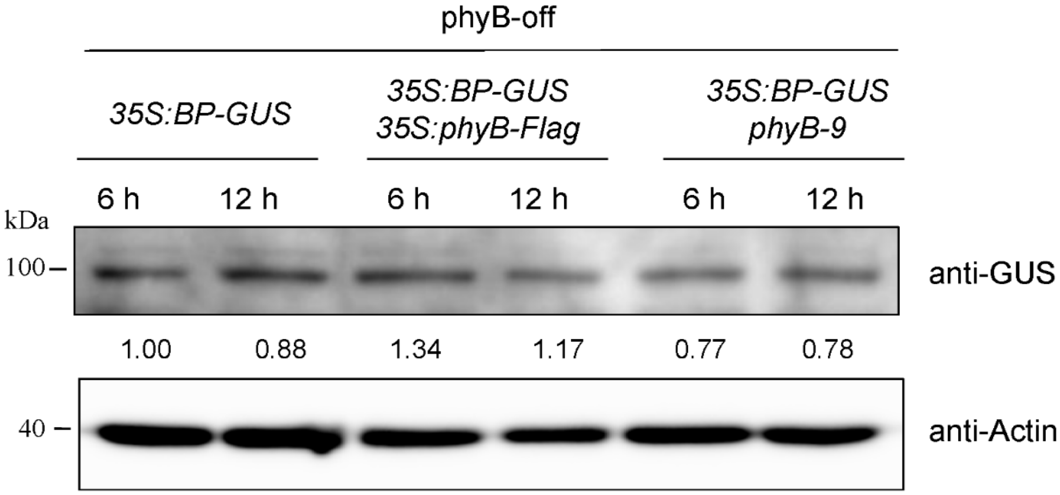

Supplemental Figure 6. Immunoblot analysis to assess the levels of BP protein in *35:BP-GUS*, *35S:BP-GUS 35S:phyB-Flag*, and *35S:BP-GUS phyB-9* seeds in the phyB-off condition. *35S:BP-GUS* and *35S:BP-GUS phyB-9* seeds were treated with FR, then incubated in the dark for 6 or 12 h, respectively. Total proteins were blotted with anti-GUS antibody. Values, which represent relative signal intensity, were normalized to the Actin control.

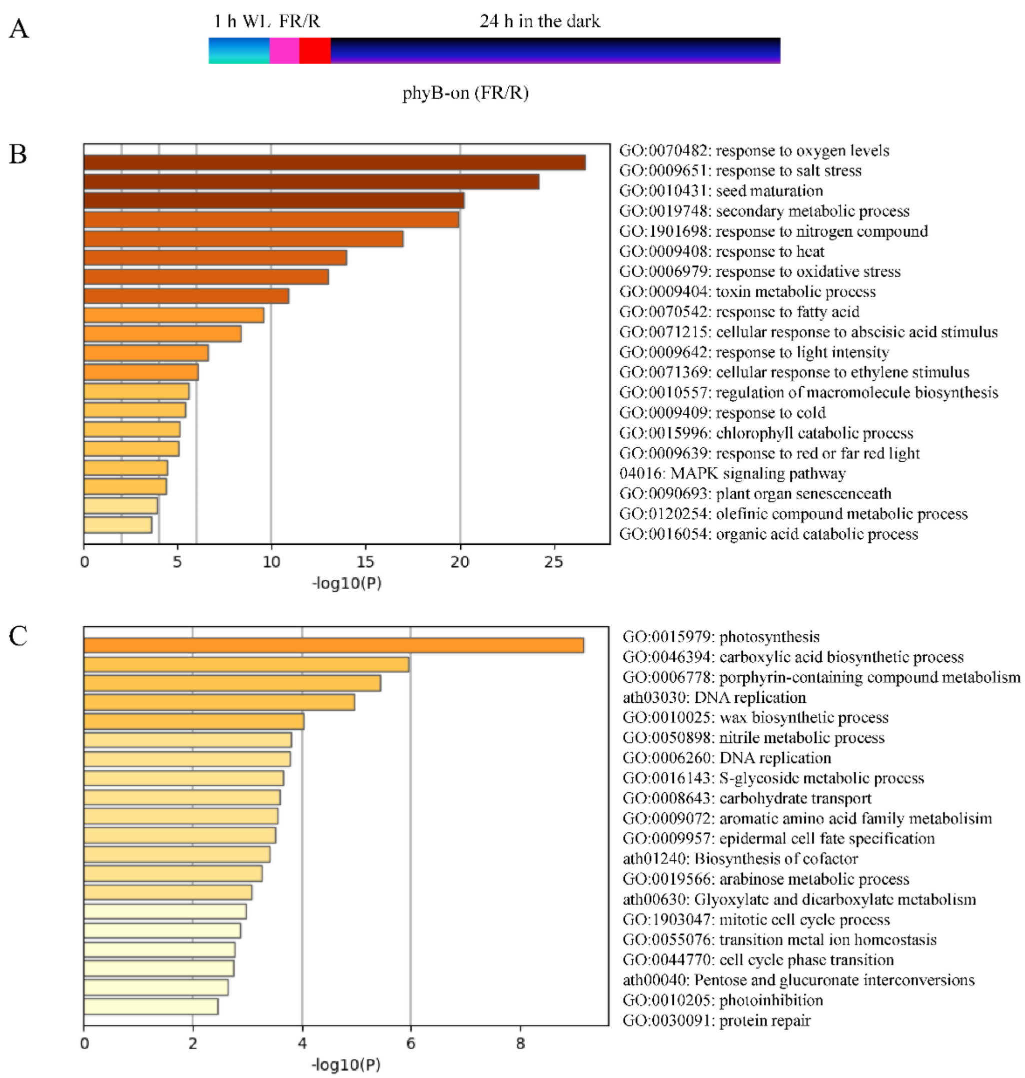

Supplemental Figure 7. Genome-wide analysis of the BP-regulated transcriptome. (A) Diagram of the phyB-on condition. FR/R, 5 min of far-red light (3.8 μmol m^{-2} s^{-1}) and then 5 min of red light (13.1 μmol m^{-2} s^{-1}). After FR/R treatment, the seeds were kept in the dark for 24 h. (B) GO biological process of the up-regulated genes in the *bp-9* mutant, as analyzed by Metascape ($P < 0.01$). (C) GO biological process of the down-regulated genes in the *bp-9* mutant, as analyzed by Metascape ($p < 0.01$).

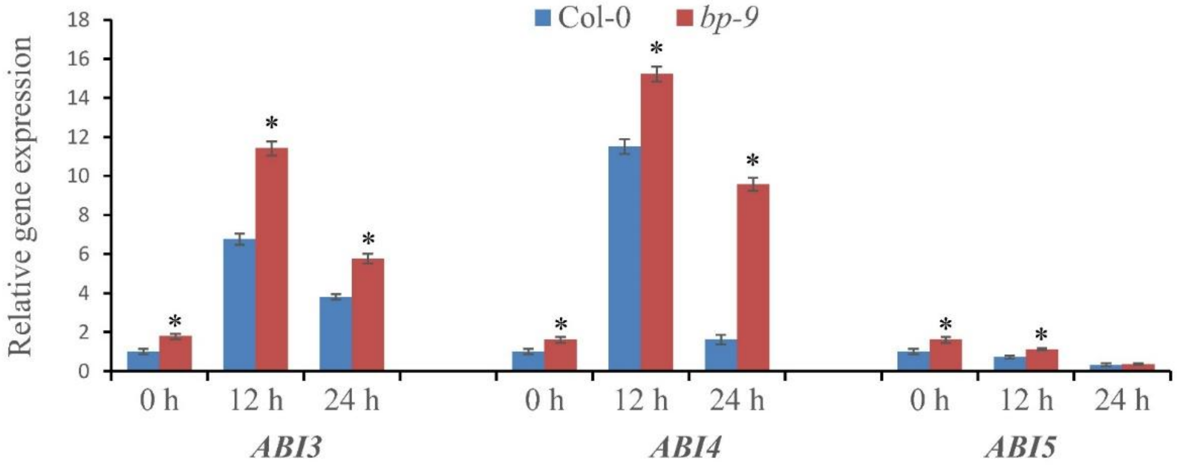

Supplemental Figure 8. RT-qPCR analysis of the expression levels of ABA signal transduction-related genes *ABI3*, *ABI4*, and *ABI5* in the *bp-9* mutant in the phyB-on condition. *PP2A* was used as the internal control. Values are shown as means ± SD (Student's *t*-test, * $p < 0.05$, n = 3).

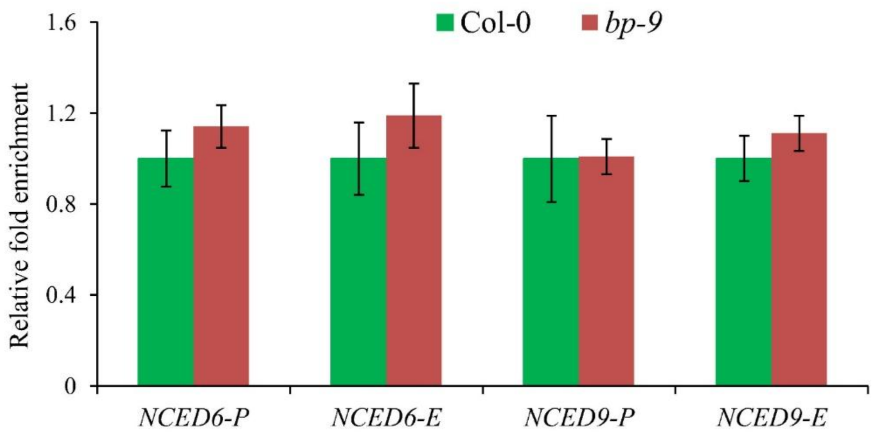

Supplemental Figure 9. ChIP-qPCR analysis of the H3K4me3 levels of *NCED* genes in imbibed Col-0 and *bp-9* mutant seeds. *ACTIN2* was used as the internal control. Values are shown as means ± SD (Student's *t*-test, * $p < 0.05$, n = 3).

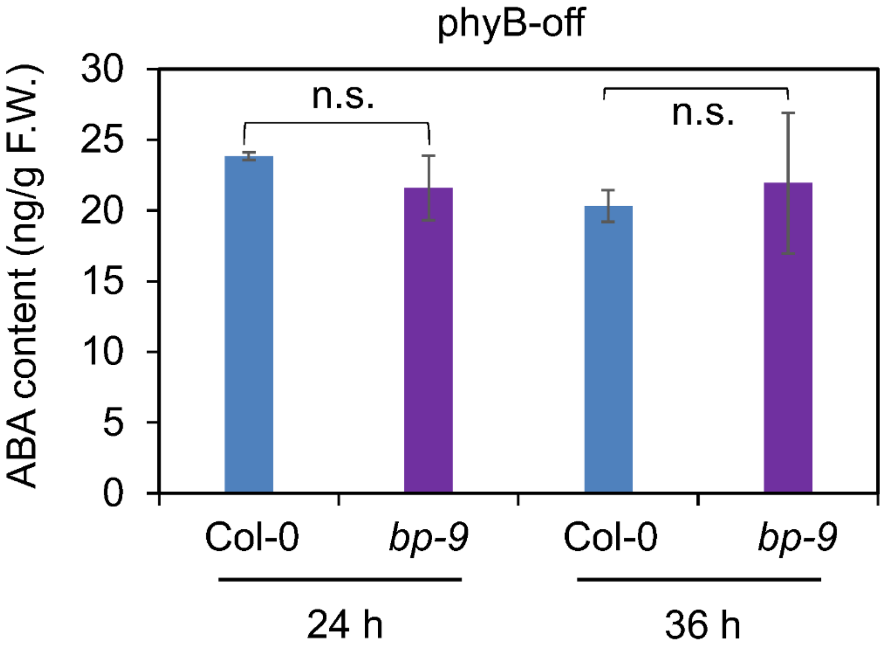

Supplemental Figure 10. Analysis of ABA levels in imbibed Col-0 and *bp-9* seeds 24 and 36 h after phyB-off treatment. Values are shown as means ± SD (Student's *t*-test, * $p < 0.05$, n=3. The n.s. means no significance.

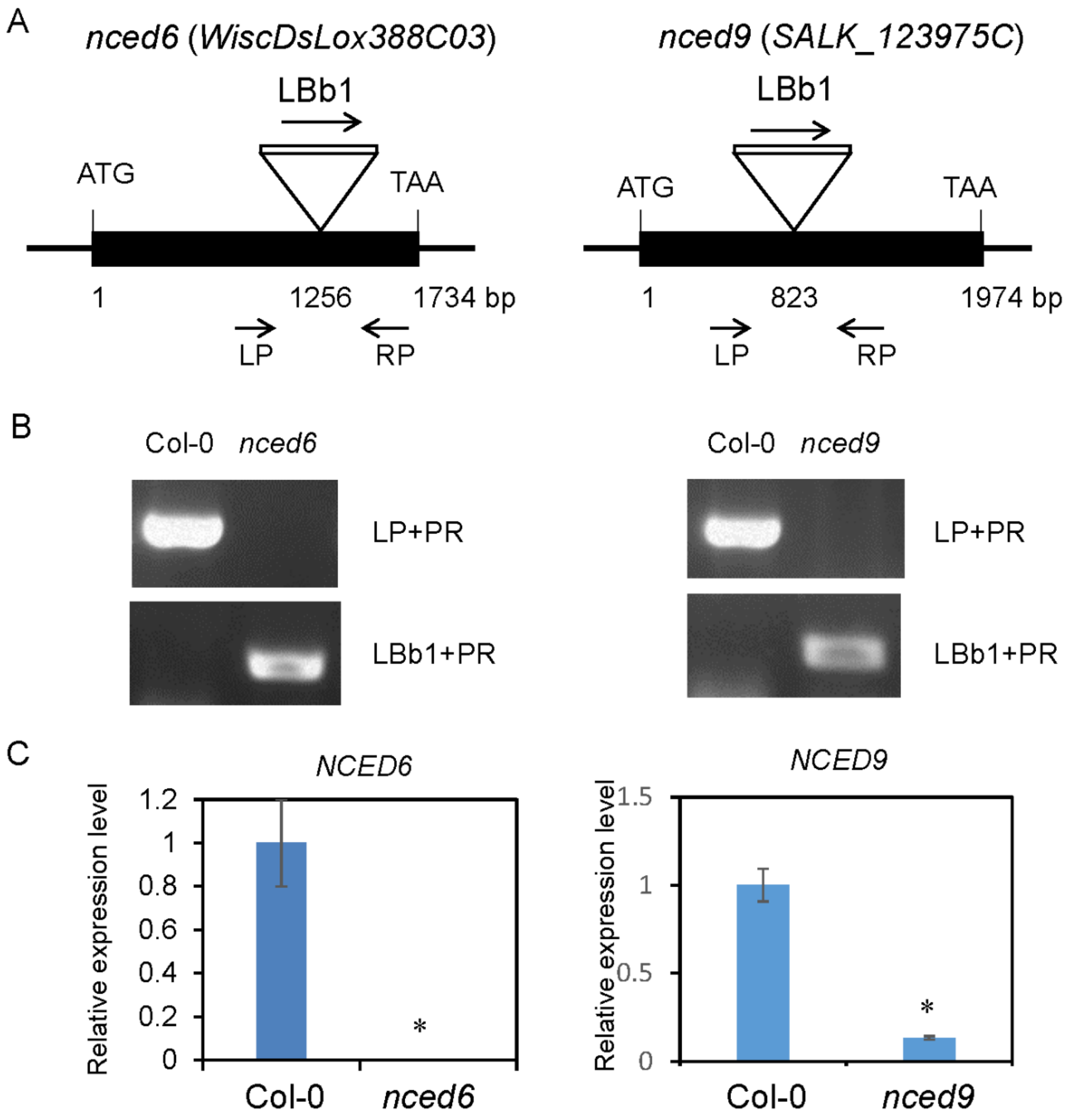

Supplemental Figure 11. Genotyping of *nced6* and *nced9* knockout mutants. (A) Schematic diagrams of the T-DNA insertion mutants of *NCED6* and *NCED9*. (B) Identification of *nced6* and *nced9* homozygous mutants by PCR analysis. (C) RT-qPCR validation of the *nced6* and *nced9* mutants. *PP2A* was used as the internal control. Values are shown as means ± SD (Student's *t*-test, * $p < 0.05$, n=3).

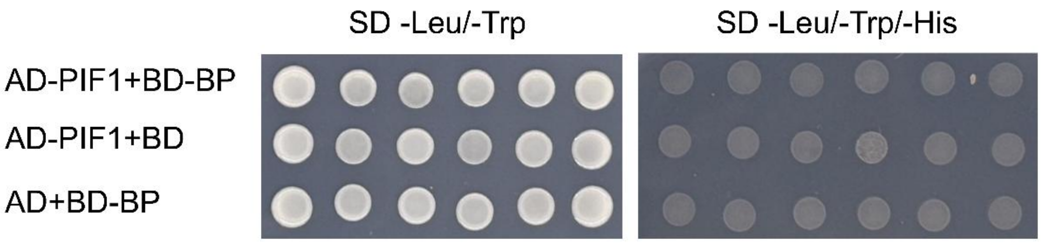

Supplemental Figure 12. Yeast two-hybrid analysis of the interaction of PIF1 with BP. PIF1 and BP fused with AD and BD vectors were co-transformed into yeast cells. The transformants were plated on SD/-Leu/-Trp and SD/-Leu/-Trp/-His dropout medium.

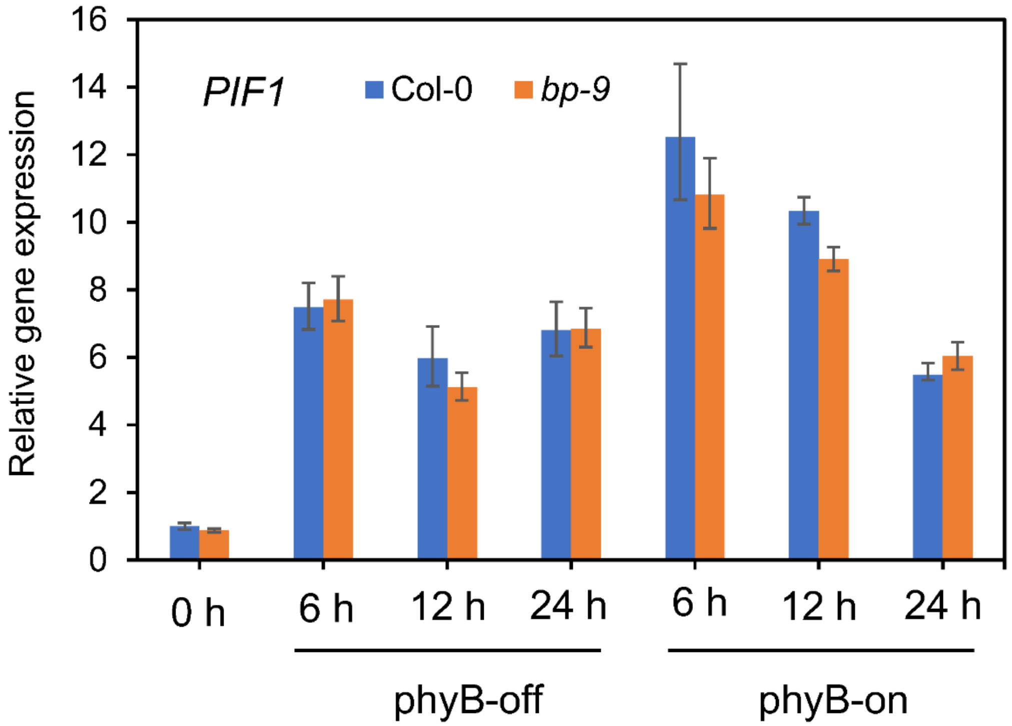

Supplemental Figure 13. RT-qPCR analysis of the expression levels of *PIF1* in imbibed Col-0 and *bp-9* seeds under phyB-on and phyB-off conditions. *PP2A* was used as internal control. Values are shown as means ± SD (Student's *t*-test, * $p < 0.05$, n=3).

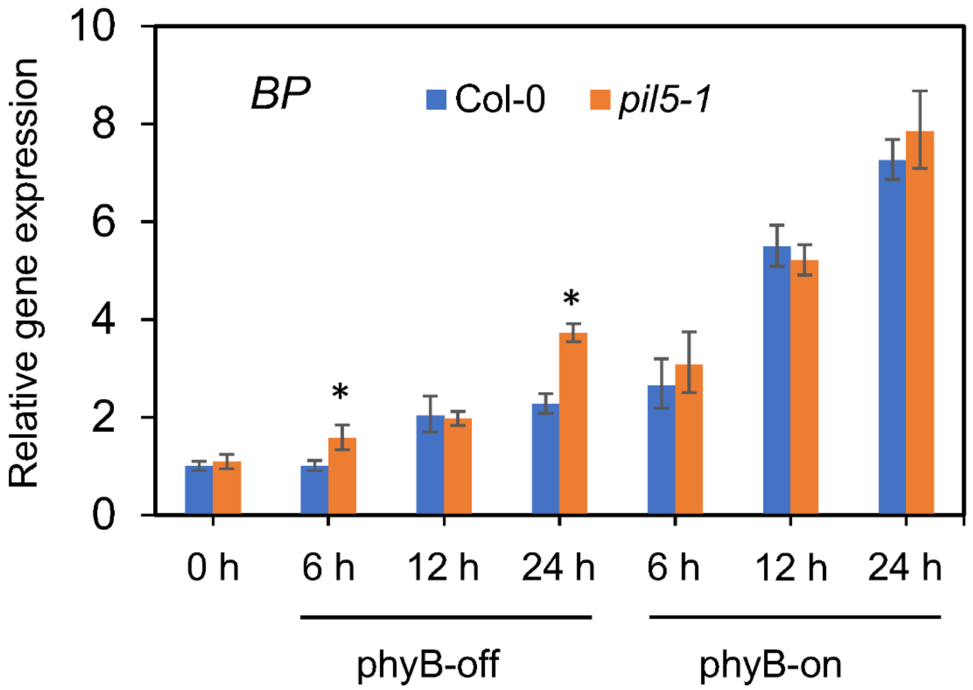

Supplemental Figure 14. RT-qPCR analysis of the expression levels of *BP* in imbibed Col-0 and *pil5-1* seeds under phyB-on and phyB-off conditions. *PP2A* was used as internal control. Values are shown as means ± SD (Student's *t*-test, * p < 0.05, n=3).

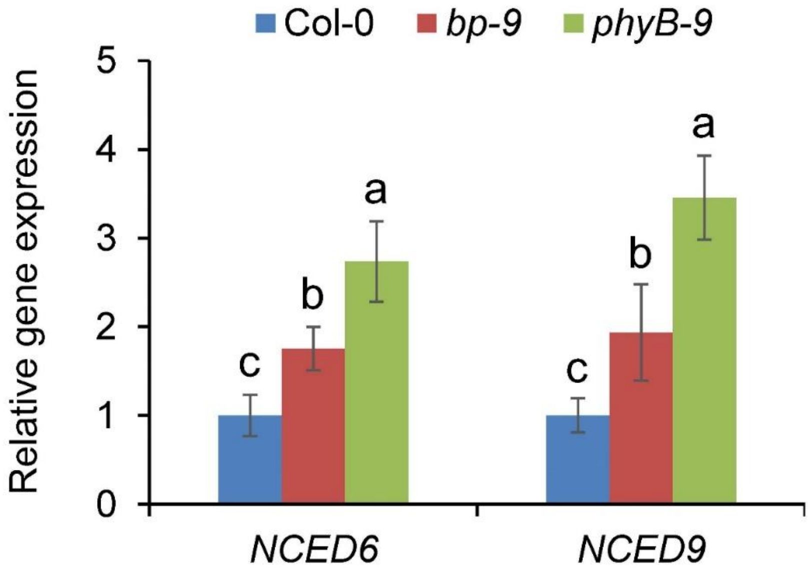

Supplemental Figure 15. RT-qPCR analysis of the expression levels of *NCED6* and *NCED9* in *bp-9* and *phyB-9* mutants in the phyB-on condition. *PP2A* was used as the internal control. Values are shown as means ± SD (n=3). The data were analyzed by one-way ANOVA followed by Tukey's HSD test. Different letters above bars indicate significant differences ($p < 0.05$).

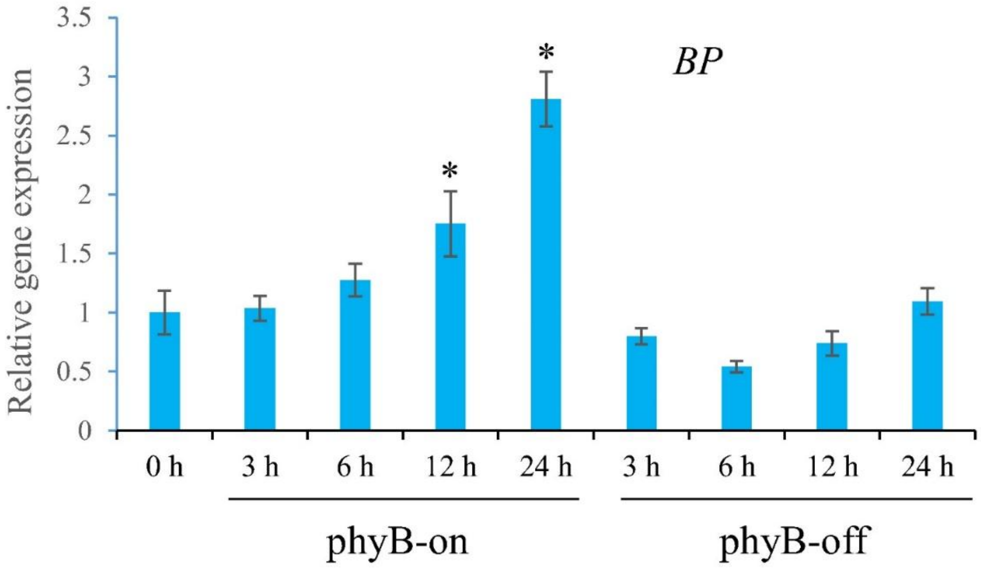

Supplemental Figure 16. RT-qPCR analysis of the expression levels of *BP* in the phyB-on and phyB-off conditions. After FR or FR/R treatments, Col-0 seeds were kept in the dark for 0, 3, 6, 12, and 24 h. *PP2A* was used the internal control. Values are shown as means ± SD (Student's *t*-test, * $p < 0.05$, n = 3).

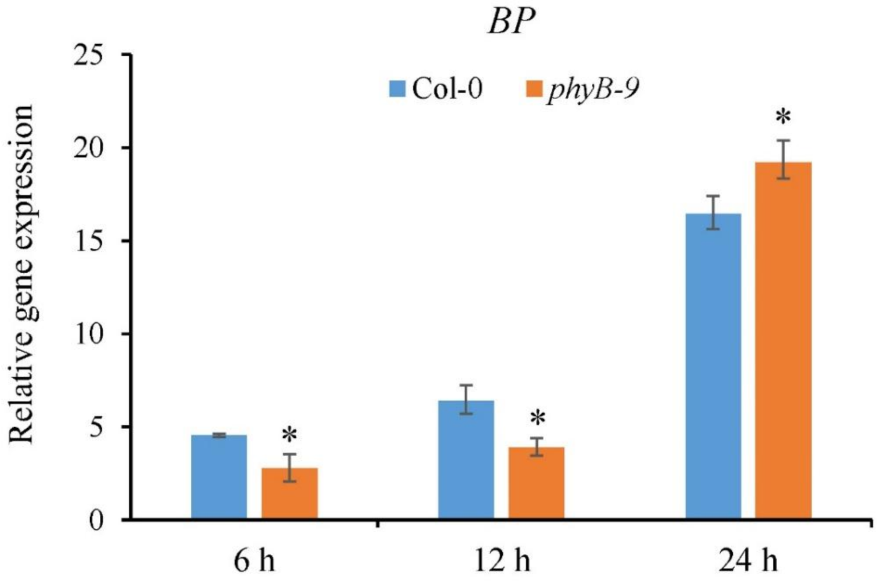

Supplemental Figure 17. RT-qPCR analysis of the expression levels of *BP* in Col-0 and the *phyB-9* mutant in the phyB-on condition. After FR/R treatments, Col-0 seeds were kept in the dark for 6, 12, and 24 h. *PP2A* was used as the internal control. Values are shown as means ± SD (Student's *t*-test, * $p < 0.05$, n = 3).

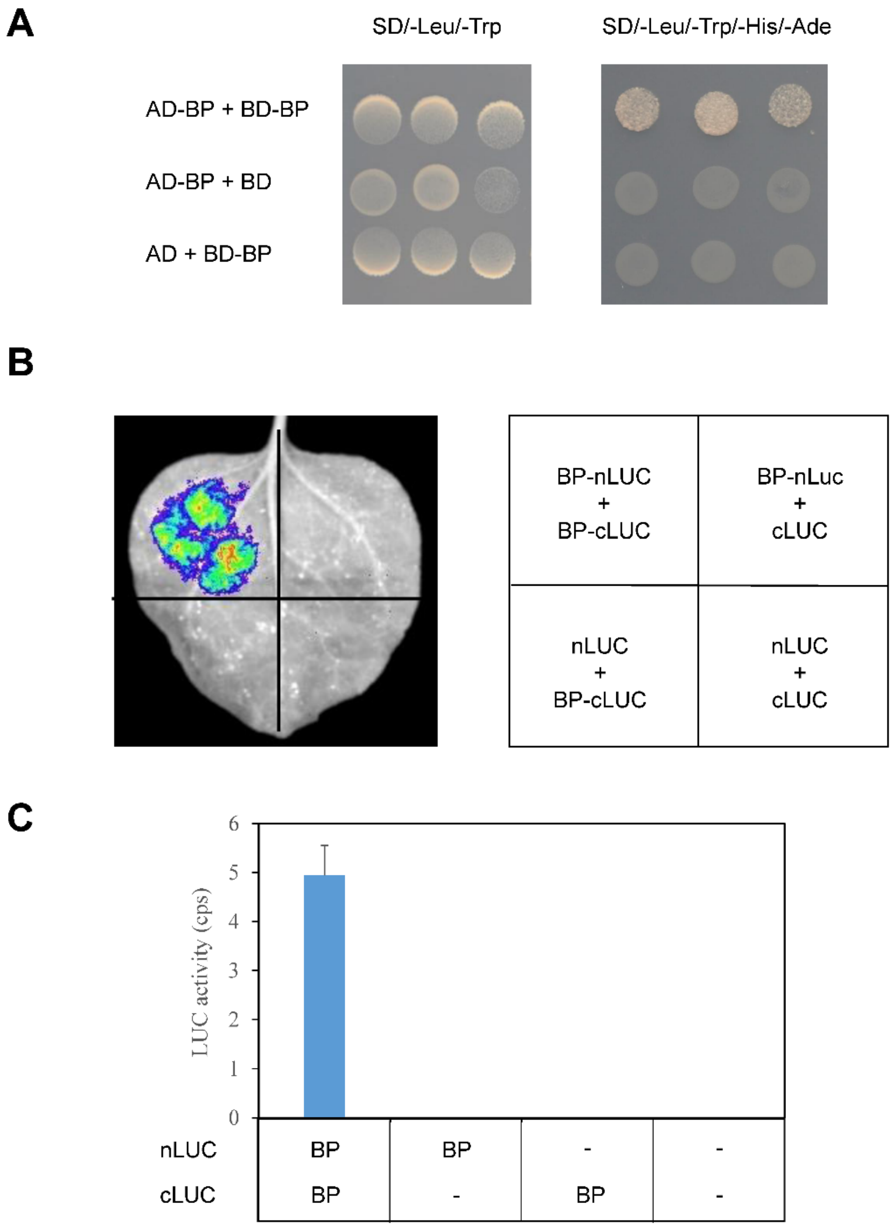

Supplemental Figure 18. BP proteins form homodimers in yeast and in plant cells. (A) Yeast two-hybrid analysis of BP-BP interaction. BP either fused with AD or BD vectors were co-transformed into yeast cells. The transformants were plated on SD/-Leu/-Trp and SD/-Leu/-Trp/-His/-Ade dropout medium. (B) LCI analysis of BP-BP interaction. BP either fused with nLUC or cLUC vectors were co-transformed into tobacco leaves and LUC luminescence signal was detected. (C) Quantification of relative LUC levels (counts per second, cps) as shown in (B). Values are shown as means ± SD.

Supplemental Table 1. LC-MS/MS analysis of the ubiquitination sites in BP protein.

Protein names	MW [kDa]	Protein score	Sequence coverage (%)	Position	Peptide score	Modified sequence	Mass error [ppm]	# PSMs
BP	45.81	14304.58	71.11	K281	22.22	AEDRELkNHLLK	0.08	1
				K135	21.31	AIHNTQEANNNNNDNVSDVEAMkAk	-3.08	1
				K137	58.83	AkIIAHPHYSTLLQAYLDcQK	1.75	1
				K207	64.82	DPELDQFMEAYcDMLVkYR	2.60	1
				K361	23.4	HWkPSEDMQFMVMDGLQHPHHAALYMDGHYMGDGPYR	2.55	1
				K156	46.63	IIAHPHYSTLLQAYLDcQkIGAPPDVVDR	0.64	1
				K287	32.01	kYSGYLSSLK	-2.94	1
				K325	45.17	LLTWWELHYkWPYPSESEK	2.56	2
				K315	29.19	QkLLTWWELHYK	-0.42	2
				K334	141.34	WPYPSESEkVALAESTGLDQK	1.11	2
				K296	64.38	YSGYLSSLkQELSK	-1.23	2

Supplement Table 2. Primers used in this study.

Primer	Sequence (5’-3’)
**Mutant screening**	
LBb1	ATTTTGCCGATTTCGGAAC
nced6-LP	TTTTGAAATGCATATGAGAGTGTG
nced6-RP	CTCTTCAGTGATAGCGCATCC
nced9-LP	GCAGCGTTTAATCAAGAATCG
nced9-RP	ATCATCGTTGGTTGATCAAGC
**Construction**	
BD-BP-pr1	GGAGGCCAGTGAATTCATGGAAGAATACCAGCATGACAAC
BD-BP-pr2	CACCCGGGTGGAATTCTGGACCGAGACGATAAGGTC
AD-PHYB-N-pr1	GCCATGGAGGCCAGTGAATTCATGGTTTCCGGAGTCGGG
AD-PHYB-N-pr2	ATGCCCACCCGGGTGGAATTCACCTAACTCATCAAT
AD-PHYB-C-pr1	GCCATGGAGGCCAGTGAATTCGAAACTGCGGAAATGGATGCG
AD-PHYB-C-pr2	ATGCCCACCCGGGTGGAATTCATATGGCATCATCAG
**qRT-PCR**	
PP2A-RT-pr1	GTGACTTGGTTGAGCATTTCACTCC
PP2A-RT-pr2	GAGCTGATTCAATTGTAGCAGCAAACT
NCED6-RT-pr1	GGTCGGATATAAATTGGGTTG
NCED6-RT-pr2	CGGGTTGGTTCTCCTGATTC
NCED9-RT-pr1	AACCGCCGCTATGGTTTTAGACG
NCED9-RT-pr2	CCAGTCACCGGAAGGTTATGCAC
ABA1-RT-pr1	GATGCAGCCAAATATGGGTCAAGG
ABA1-RT-pr2	GCCATTGCATGGATAATAGCGACTC
ABA2-RT-pr1	AGAGGTGTTTGCATGATTCCTGAGC
ABA2-RT-pr2	TCCAGTGATCAATGCCACTTTACCC
ABI3-RT pr1	CTTGAAGCAAAGCGACGTGG
ABI3-RT-pr2	TGTCTTACTTTAACCCCTCGTAT
ABI4-RT-pr1	TCCGCTCAACGCAAACG
ABI4-RT-pr2	TTGTCGAACGCCACGGTA
ABI5-RT-pr1	CAATAAGAGAGGGATAGCGAACGAG
ABI5-RT-pr2	CGTCCATTGCTGTCTCCTCCA
BP-RT-pr1	GGGAAGAGTGACAATATGGG
BP-RT-pr2	TATGGACCGAGACGATAAGG
**ChIP-qPCR**	
TA3-ChIP-F	GATTCTTACTGTAAAGAACATGGCATTGAGAGA
TA3-ChIP-R	TCCAAATTTCCTGAGGTGCTTGTAACC
ACTIN2-ChIP-F	GCACCCTGTTCTTCTTACCG
ACTIN2-ChIP-R	AACCCTCGTAGATTGGCACA

NCED6-P1-F	GATTCGACACCTATTTAGGTTC
NCED6-P1-R	ATGTTCTCTACTATAATGTGGAGT
NCED6-P2-F	GAAGTGGTTGGTCAGATTCC
NCED6-P2-R	TACCGGCAGCTGTAACTAAC
NCED6-P3-F	TCGGACGGTTCGGATTCG
NCED6-P3-R	TCATGAATCATCGTTGGTTCAGG
NCED9-P1-F	AAGCAAGCGCGTAAGACATAC
NCED9-P1-R	CTGACGAAGAATACACACAACAC
NCED9-P2-F	CGCAGCGTTTAATCAAGAATCG
NCED9-P2-R	TGAAGTTGAGAAAGTTCGGTCG
NCED9-P3-F	ACGGAGGAGAGCCTCTGTTTC
NCED9-P3-R	CGGCGTTTATGAGTTGGAGTTCC
**EMSA analysis**	
NCED6-F-biotin	TAGCCGGACATCATTTATTTGACGGTGACGGAATGATTCACGCCGTTA
NCED6-R-biotin	TAACGGCGTGAATCATTCCGTCACCGTCAAATAAATGATGTCCGGCTA
NCED6-M-F	TAGCCGGACATCATTTATTAAAAGGAAAAGGAATGATTCACGCCGTTA
NCED6-M-R	TAACGGCGTGAATCATTCCTTTTCCTTTTAATAAATGATGTCCGGCTA
NCED6-M-F-biotin	TAGCCGGACATCATTTATTAAAAGGAAAAGGAATGATTCACGCCGTTA
NCED6-M-R-biotin	TAACGGCGTGAATCATTCCTTTTCCTTTTAATAAATGATGTCCGGCTA
NCED9-F-biotin	CATTACACTTTTTTAAAATGACTGGAATGCATGACGTTTGGGCAC
NCED9-R-biotin	GTGCCCAAACGTCATGCATTCCAGTCATTTTAAAAAAGTGTAATG
NCED9-M-F	CATTACACTTTTTTAAAAAAAATGGAATGCAAAAAGTTTGGGCAC
NCED9-M-R	GTGCCCAAACTTTTTGCATTCCATTTTTTTTAAAAAAGTGTAATG
NCED9-M-F-biotin	CATTACACTTTTTTAAAAAAAATGGAATGCAAAAAGTTTGGGCAC
NCED9-M-R-biotin	GTGCCCAAACTTTTTGCATTCCATTTTTTTTAAAAAAGTGTAATG
NCED6-F-cold	TAGCCGGACATCATTTATTTGACGGTGACGGAATGATTCACGCCGTTA
NCED6-R-cold	TAACGGCGTGAATCATTCCGTCACCGTCAAATAAATGATGTCCGGCTA
NCED9-F-cold	CATTACACTTTTTTAAAATGACTGGAATGCATGACGTTTGGGCAC
NCED9-R-cold	GTGCCCAAACGTCATGCATTCCAGTCATTTTAAAAAAGTGTAATG
**Dual-luciferase reporter analysis**	
SK-BP-F	TAGAACTAGTGGAT ATGGAAGAATACCAGCATGACA
SK-BP-R	CGGTATCGATAAGCTTTTATGGACCGAGACGATAAGGT
NCED9pro-F	TATAGGGCGAATTGGGTACCGTGAAATTACTAAGTACTGTAGCAAGTGTA
NCED9pro-R	TTGGCGTCTTCCATGGTCACGCTACTATTTTCTCATCTTTTAACAC
NCED6extron-F	TATAGGGCGAATTGGGTACCTCGAAATTCTGTATTCTGAAAACATTCGAC
NCED6extron-R	TTGGCGTCTTCCATGGTTGAACAAGCCGGTTTGTTTTAGTGTACCG
